# Deprivation of methionine inhibits osteosarcoma growth and metastasis via *C1orf112*-mediated regulation of mitochondrial functions

**DOI:** 10.1038/s41419-024-06727-1

**Published:** 2024-05-20

**Authors:** Xindan Zhang, Zhenggang Zhao, Xuepeng Wang, Shiwei Zhang, Zilong Zhao, Wenbin Feng, Lijun Xu, Junhua Nie, Hong Li, Jia Liu, Gengmiao Xiao, Yu Zhang, Haomiao Li, Ming Lu, Jialuo Mai, Sujin Zhou, Allan Z. Zhao, Fanghong Li

**Affiliations:** 1 The School of Biomedical and Pharmaceutical Sciences, Guangdong University of Technology, Guangzhou, China; 2 South China University of Technology School of Medicine, Guangzhou, China; 3 Biomedical Laboratory, Guangzhou Jingke Life Science Institute, Guangzhou, China; 4 Department of Orthopedic Oncology, Guangdong Provincial People’s Hospital Affiliated to South China University of Technology School of Medicine, Guangzhou, China; 5 Department of Musculoskeletal Oncology, Center for Orthopedic Surgery, The Third Affiliated Hospital of Southern Medical University, Guangzhou, China; 6 Guangzhou Sinogen Pharmaceutical Co., Ltd., Guangzhou, Guangdong Province China

**Keywords:** Bone cancer, Metastasis

## Abstract

Osteosarcoma is a malignant bone tumor that primarily inflicts the youth. It often metastasizes to the lungs after chemotherapy failure, which eventually shortens patients’ lives. Thus, there is a dire clinical need to develop a novel therapy to tackle osteosarcoma metastasis. Methionine dependence is a special metabolic characteristic of most malignant tumor cells that may offer a target pathway for such therapy. Herein, we demonstrated that methionine deficiency restricted the growth and metastasis of cultured human osteosarcoma cells. A genetically engineered *Salmonella*, SGN1, capable of overexpressing an L-methioninase and hydrolyzing methionine led to significant reduction of methionine and S-adenosyl-methionine (SAM) specifically in tumor tissues, drastically restricted the growth and metastasis in subcutaneous xenograft, orthotopic, and tail vein-injected metastatic models, and prolonged the survival of the model animals. SGN1 also sharply suppressed the growth of patient-derived organoid and xenograft. Methionine restriction in the osteosarcoma cells initiated severe mitochondrial dysfunction, as evident in the dysregulated gene expression of respiratory chains, increased mitochondrial ROS generation, reduced ATP production, decreased basal and maximum respiration, and damaged mitochondrial membrane potential. Transcriptomic and molecular analysis revealed the reduction of *C1orf112* expression as a primary mechanism underlies methionine deprivation-initiated suppression on the growth and metastasis as well as mitochondrial functions. Collectively, our findings unraveled a molecular linkage between methionine restriction, mitochondrial function, and osteosarcoma growth and metastasis. A pharmacological agent, such as SGN1, that can achieve tumor specific deprivation of methionine may represent a promising modality against the metastasis of osteosarcoma and potentially other types of sarcomas as well.

## Introduction

Osteosarcoma is the most common primary bone malignancy with a high proclivity for local invasion and metastasis [1, 2]. Despite the fact that combining surgery and chemotherapy has greatly improved the outcomes of osteosarcoma patients [3], roughly 30–50% of osteosarcoma patients experience recurrence, which mostly occurs 2–3 years after the prospective treatment, and less than 30% of recurrent patients could survive for more than five years [4]. Importantly, the 5-year survival rate for patients with metastatic osteosarcoma remains at only 10–30% [5]. Therefore, there is an urgent need to develop novel therapeutic strategies to reduce the occurrence of metastasis and improve the overall survival in osteosarcoma patients.

We have been focusing on some of the unique features of cancer cell metabolism. In contrast to the biochemistry of non-cancerous cells, methionine deprivation can powerfully modulate the processes of DNA methylation, cell cycle transition, polyamines, and antioxidant synthesis of tumor cells [6, 7]. Under the stress of methionine restriction, human non-cancerous cells can take advantage of “methionine salvage biochemical pathway”, mainly involving the activity of methylthioadenosine phosphorylase (MTAP) as a substrate, to “salvage” the deficiency of methionine [6]. However, in almost all types of human cancer, particularly osteosarcoma cells, the MTAP activity is severely lacking, rendering the salvage pathway deficient [8–10]. Therefore, the absolute-dependency on exogenous supply of L-methionine for the growth and proliferation of tumors is a pivotal biochemical criterion for various human cancers [11].

The use of bacteria as bio-therapeutic agents for cancer treatment has a long and interesting history [12]. Over the last few decades, numerous studies have emerged and re-invigorated the field of bacteria-based bio-therapeutics for the treatment of cancer [13, 14]. Indeed, several bacterial genera have been evaluated in pre-clinical cancer models, including *Bifidobacterium*, *Clostridium*, and *Salmonella enterica serovar Typhimurium* [15–17]. As evidenced by the volume of studies in the current literature, *Salmonella* is by far the most extensively evaluated and characterized bacterial genus currently being explored as a potential modality for bio-therapeutic agent against cancer [18, 19]. However, the outcomes from the clinical trials indicate that the bacteria alone are not sufficient to stop the progression of cancer [20]; additional engineering of cancer-killing (e.g., TNF-α) or induction of cancer-killing (such as antigen) gene is required for the development of successful oncology drug [21].

In our latest study, we have described the development of an attenuated *Salmonella*-based agent, SGN1, that can target methionine metabolism [22]. SGN1 is a genetically modified strain of attenuated *Salmonella typhimurium* (VNP20009) that can overexpress an L-methioninase—a pyridoxal phosphate-dependent enzyme that catalyzes the γ-elimination of amino group of L-methionine to methanethiol, α-ketobutyrate, and ammonia [23, 24]. SGN1 can target, and preferentially replicate in the tumors, which in turn specifically deprive them of an essential amino acid, methionine, via the enzymatic activity of L-methioninase. Currently, SGN1 has entered the trials for clinical evaluation in the US (NCT05103345 & NCT05038150). However, systematic pharmacology study of SGN1 against osteosarcoma, particularly the ability to restrain metastasis, is still required to provide the basis for future clinical trials in osteosarcoma.

Herein, we provide the evidence that methionine deprivation, attained by either methionine depletion or SGN1, can sharply inhibit the growth and metastasis of osteosarcoma cells in pre-clinical cellular and several human osteosarcoma cell-derived models (subcutaneous model, metastatic model, orthotopic model, as well as patient-derived organoid or xenograft models). Mechanistically, we demonstrate that methionine deprivation controls the growth and metastasis of osteosarcoma through *C1orf112* -mediated mitochondrial dysfunction.

## Results

### The inhibitory effects of methionine deprivation or SGN1 on osteosarcoma growth

**Fig. 1:**
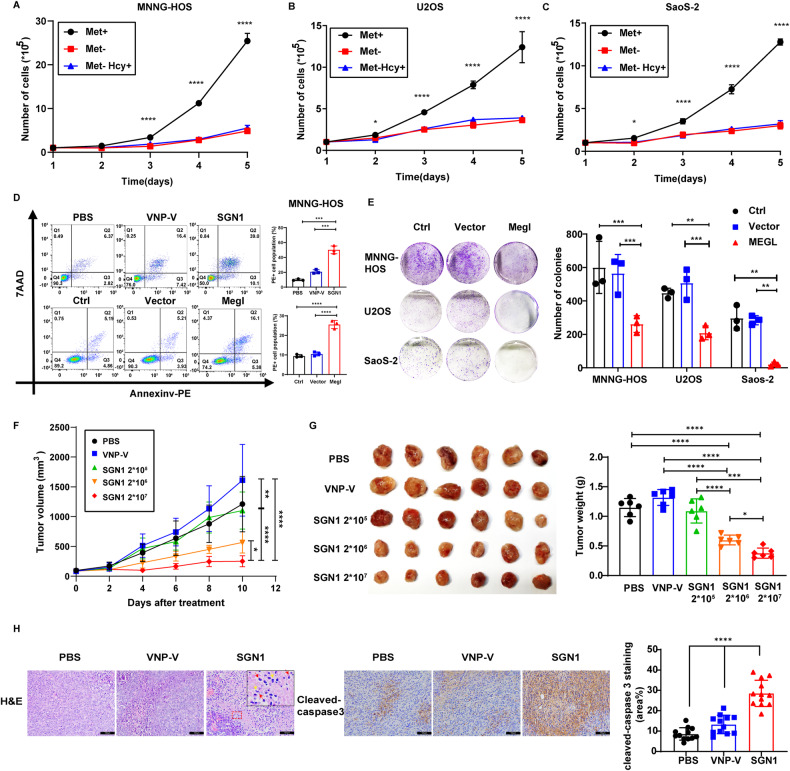
The effects of methionine deficiency and dose-dependent growth inhibition of SGN1 on osteosarcoma cells. **A**–**C** The effects of methionine deficiency on osteosarcoma cell proliferation were assessed by cell counting assay. Osteosarcoma cells were cultured in medium without methionine (MET) for 5 days. Homocysteine (HCY) supplementation cannot rescue the cell proliferation caused by MET deficiency (*n* = 3). **D** The apoptosis-inducing effect of different types of bacteria or *L-methioninase* (Megl) was investigated by flow cytometric analysis of MNNG-HOS cells stained with Annexin V and 7-AAD (*n* = 3). **E** Left panel: colony formation image of MNNG-HOS, U2OS, SaoS-2 cells overexpressing L-methioninase; right panel: quantification of colony formation assay (*n* = 3). **F** Tumor growth curve of BALB/c nude mice with MNNG-HOS xenografts after received a single intratumoural injection of vehicle (PBS), VNP-V (2 × 10^6^ CFU/ mouse), SGN1(2 × 10^5^, 2 × 10^6^, and 2 × 10^7^ CFU/ mouse) (*n* = 30 total, 6 mice per group). **G** Left panel: the image of tumors obtained at the end of treatment; right panel: the tumor weight from the mice bearing xenografts of MNNG-HOS cells at the end of treatment. **H** Left panel: representative microphotographs of H&E and Cleaved-Caspase3 immunohistochemical staining of tumor tissue of nude mice treated with 2 × 10^6^ CFU/ mouse SGN1. Scale bars:100 µm; right panel: quantitative analysis of Cleaved-Caspase3 positive cells. At higher magnification, the signs of both apoptotic (red arrows; cytoplasmic condensation, pyknotic and fragmented nuclei, and rounded hyperchromatic apoptotic bodies) and necrotic (yellow arrows; nuclear swelling and pale cytoplasm, karyorrhexis and increased cell volume) cells can be observed. Data shown as mean ± SD. In (**A**, **B**, **C**, **D**, **E**, **G**, **H**), the *p* values are derived from one-way ANOVA analysis followed by Tukey’s multiple comparison test. In (**F**), the *p* values are derived from the two-way ANOVA analysis followed by Tukey’s multiple comparison test. The symbols, *P < 0.05, **P < 0.01, ***P < 0.001, ****P < 0.0001, respectively.

Although the dependency of many cancer cells on methionine has been established [25], we still made sure that all three human osteosarcoma cell lines used in this study rely heavily on methionine for growth, as evidenced by the cultured cells in which methionine was deliberately stripped from the medium (Fig. 1A–C). With this knowledge in hand, we further evaluated the impact of SGN1, the engineered *Salmonella* overexpressing methionine-hydrolyzing enzyme (*L-methioninase*), on the cultured osteosarcoma cells. Co-culture of SGN1 generated cytotoxic effects on osteosarcoma cells in a dose-dependent manner (Fig. S1A–C). We also analyzed cell death after incubating SGN1 or the control *Salmonella* (VNP-V) for 5 h. SGN1 drastically increased the percentage of all dead cell population (Fig. 1D and S1D, E), even far above those induced by the control VNP-V, suggesting that the cell death-inducing effect of SGN1 was primarily due to the methionine-hydrolyzing activity of the engineered methioninase. Indeed, LC-MS/MS assay further revealed a significant reduction of cellular methionine content after SGN1 treatment (Fig. S1F). In order to corroborate such findings, we transfected the osteosarcoma cells with the cDNA encoding the *L-methioninase*. Cell clone formation clearly demonstrated that L-methioninase overexpression led to sharp reductions of cell growth in all three osteosarcoma cell lines (Fig. 1E), which was also accompanied by a large drop of cellular methionine content (Fig. S1G). The similar induction of cell death (apoptosis and necrosis) of *L-methioninase* with respect to SGN1 was observed by flow cytometry using Annexin V/7-amino-actinomycin staining. (Fig. 1D and S1D, E). To further delineate the extent of apoptosis and necrosis following the deprivation of methionine by L-methioninase, we evaluated the impact of apoptosis inhibitor Z-VAD-FMK (a cell-permeable pan-caspase inhibitor) and necrosis inhibitor, Necrostatin-1 (Nec-1, a potent and specific small-molecule inhibitor of receptor-interacting serine/threonine-protein kinase 1 (RIPK1, aka RIP1)), on the MNNG-HOS cells overexpressing the *L-methioninase*. As expected, overexpression of *L-methioninase* led to almost complete inhibition of cell proliferation; Z-VAD-FMK treatment restored about 80% and Nec-1 40% of the cell viability (Fig. S1H). The expression of *L-methioninase* led to an increase in cleaved-PARP and a decrease in Bcl-2 expression (Fig. S1I). Thus, methionine deprivation induces primarily apoptosis and, to a lesser extent, necrosis in osteosarcoma cells.

The findings from the cultured osteosarcoma cells prompted us to further investigate the impact of SGN1 on the subcutaneous xenografts derived from the MNNG-HOS cell line. After the tumors had grown to the expected size, the mice were randomly assigned to receive a single intra-tumoral injection of SGN1 (2 × 10^5^, 2 × 10^6^, and 2 × 10^7^CFU/mouse), VNP-V (the control *Salmonella*, 2 × 10^6^ CFU/mouse), and PBS. SGN1 inhibited the growth of subcutaneous xenograft in a dose-dependent manner, with significantly smaller tumor volume and weight in the SGN1 groups than in the VNP-V and PBS groups 10 days post treatment (Fig. 1F–G). Hematoxylin and eosin (H&E) and immunohistochemical staining revealed that the tumor tissues treated with SGN1 (at 2 × 10^6^ or 2 × 10^7^ CFU) had widespread cell death and more intense cleaved caspase-3 staining than the control samples (Fig. 1H). Importantly, consistent with the results from the cellular studies described above, LC-MS/MS analysis of the tumors revealed a significant decrease in methionine, S-adenosyl methionine (SAM), and S-Adenosyl-L-homocysteine (SAH) in the SGN1-treated group versus the two controls (Fig. S1J). Combined together, these data showed that SGN1 could inhibit the growth of human osteosarcoma cell-derived xenografts in a dose-dependent manner, accompanied by the reduction of methionine (or methylation donors) in the tumor tissues.

### Tumor-suppressive activity of SGN1 in osteosarcoma patient-derived organoid (PDO) and patient-derived xenograft (PDX) models

**Fig. 2:**
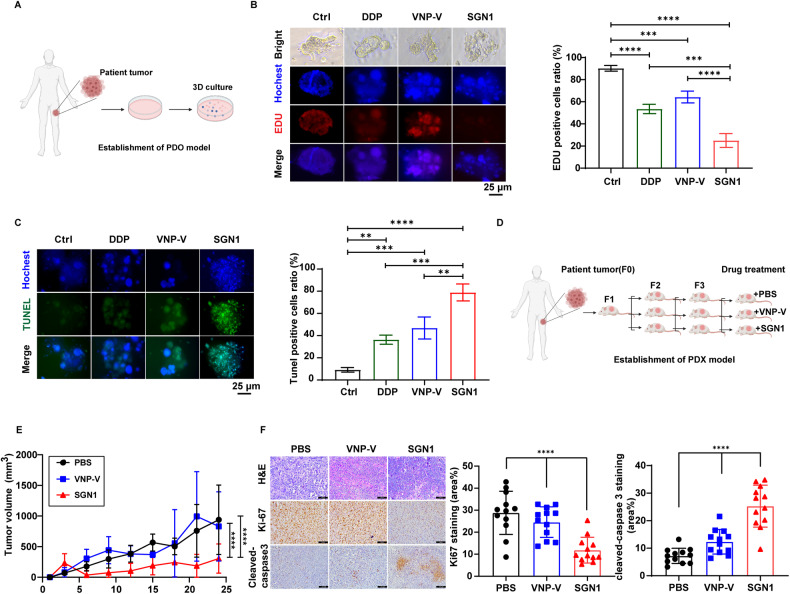
Tumor-suppressive activity of SGN1 in osteosarcoma PDO and PDX models. **A** Schematic of osteosarcoma PDO models. **B** Left panel: representative images of osteosarcoma PDO models labeled with EDU (red), fluorescent Hochest (blue). Scale bars: 25 µm; right panel: quantitative analysis of EDU positive cells. **C** Left panel: representative images of osteosarcoma PDO models labeled with Tunel (Green). Scale bars: 25 µm; right panel: quantitative analysis of Tunel (Green) positive cells **D** Schematic of osteosarcoma PDX model. Primary human osteosarcoma tissue was implanted into an NOD-SCID mice (F1) and expanded into larger cohort of mice (F2&F3), and then collected for histological assessment and in vivo testing. **E** Tumor growth curves of the PDX tumors volume (*n* = 6). **F** Left panel: representative microphotographs of H&E, immunofluorescence staining for Ki-67 and Cleaved-Caspase3 of tumor from PDXs mice treated with 2 × 10^6^ CFU/ mouse SGN1. Scale bars: 100 µm; right panel: quantitative analysis of Ki-67 and Cleaved-Caspase3 positive cells. Data shown as mean ± SD. In (**B**, **C**, **F**), the *p* values are derived from one-way ANOVA analysis followed by Tukey’s multiple comparison test. In (**E**), the *p* values are derived from the two-way ANOVA analysis followed by Tukey’s multiple comparison test. *P < 0.05, **P < 0.01, ***P < 0.001, ****P < 0.0001, respectively.

To further validate the observations made in the cellular model and xenograft animal model, we want to study the effect of metabolic deprivation of methionine achieved by pharmacological treatment with SGN1 in the models approximating the clinical status of osteosarcoma. Patient-derived organoids (PDO) is considered as high-quality drug screening and evaluation models [26]. We harvested the viable tumor tissues from the primary osteosarcoma lesions of a 5-years-old female patient and a 19-years-old male patient to establish PDO models by 3D culture (Material & Methods, Fig. 2A). In testing the sensitivity of such cultured PDOs to four clinically approved front line chemo drugs, we found that the PDOs derived from both patients were found to be most sensitive to cisplatin (DDP) and least sensitive to adriamycin (ADM) (Fig. S2A). Such screening helped us to establish the use of cisplatin as the positive drug control. SGN1 or VNP-V was co-imbedded with an organoid in matrigel at a low level of 1.5 × 10^4^CFU/well (a mere 5:1 ratio of bacterial to cancer cells, see Material & Methods). The concentration of DDP was selected as 4 µM, which is commonly used in other pharmacology screening studies [27]. Following the co-incubation, the SGN1-treated group showed the most dramatic changes in morphology and spheroid growth with surface pyknosis and emergence of punctuation, and the organoid mass lysed into individual cells (Fig. 2B). Staining with 5-ethynyl-2’-deoxyuridine (EdU) clearly showed that the SGN1 treatment led to a significant drop in the proliferation rate of cancer cells within the organoids relatively to the negative control (DMSO) and VNP-V groups (Fig. 2B). Although DDP-treatment also generated substantial inhibitory effect on the growth of the organoids, it failed to reach the same level achieved by SGN1 (Fig. 2B). SGN1 treatment also triggered cellular death in the co-cultured organoid, as reflected in the sharp elevation of TUNEL (terminal deoxynucleotidyl transferase dUTP nick end labeling)-stained positive cells (green fluorescence) (Fig. 2C). The cell death induced by DDP was far below that by SGN1, indicating that, unlike DDP, the strong killing ability of SGN1 involves both the suppression of cell proliferation and stimulation of cell death in osteosarcoma organoids.

Patient-derived xenograft (PDX) are widely used as preclinical models for approximating clinical tumor status and for evaluating oncology drug efficacy [28]. The viable tumor tissue taken from the primary lesion of the 5-years old osteosarcoma patient was engrafted into immunocompromised mice (Fig. 2D). The PDXs model exhibited histological characteristics similar to those of the primary tumor (Fig. S2B, C), suggesting that the model was successfully generated. Compared with those in the PBS or VNP-V-treated group, intra-tumoral delivery of SGN1 at a dose of 2 × 10^6^ CFU/mouse led to a drastic retardation of tumor growth, whereas the same dose of VNP-V control had little effect on the PDX-tumor model (Fig. 2E). During the treatment, there were no significant changes in body weight for all groups (Fig. S2D). Immunohistochemistry of PDX tumors showed that SGN1 treatment significantly decreased Ki-67 expression, while the level of the cleaved caspase-3 was elevated (Fig. 2F). Both the strong TUNEL staining in the PDOs model and the increased staining of the cleaved caspase-3 revealed a robust apoptosis-inducing effect of SGN1. With the data derived from both PDOs and PDXs experiments, SGN1 unequivocally showed its strong inhibitory effect on clinically-approximated cancer models.

### The inhibitory effect of SGN1 on the distal metastasis of osteosarcoma

**Fig. 3:**
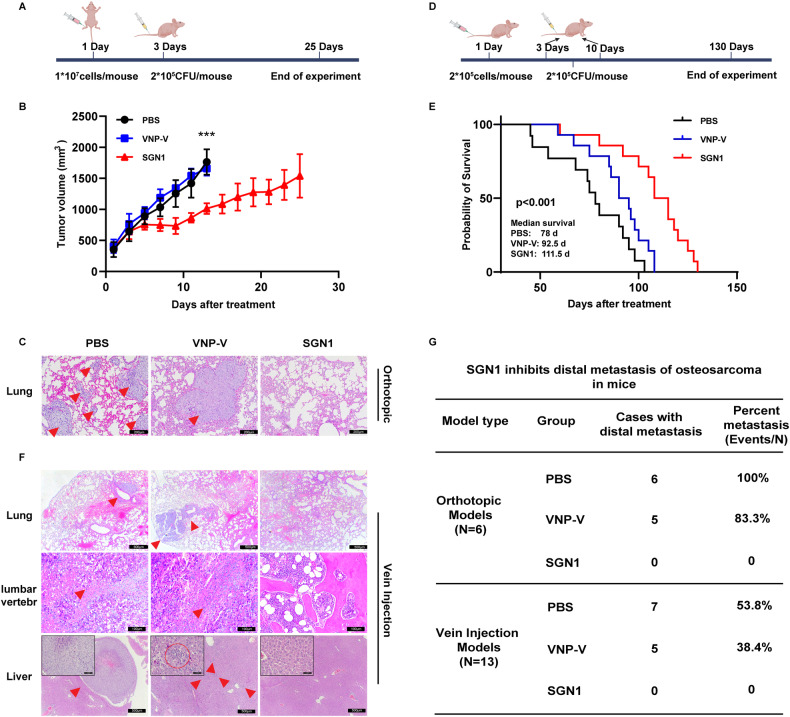
The inhibitory effect of SGN1 on the distal metastasis of osteosarcoma. **A** The designed treatment and evaluation schedule of the in situ osteosarcoma models. **B** Tumor growth curves of *Balb/c nu/nu* mice with MNNG-HOS orthotopic models after treatment with SGN1 (*n* = 6 per group). **C** H&E staining of lung metastases (triangle) at the end of the treatment in orthotopic models. Scale bars: 200 µm. **D** The designed treatment and evaluation schedule of the caudal vein metastasis model. **E** Kaplan–Meier survival analysis of survival rate of the vein injection models (*n* = 13 per group). **F** H&E staining of lung metastases (triangle), lumbar vertebra metastases (triangle), and liver metastases (triangle) at the end of treatment in vein injection models. Scale bars: 200 µm. **G** The percent distal metastases mice in each group of orthotopic models and vein injection models. Data shown as mean ± SD. In (**B**), the *p* values are derived from two-way ANOVA analysis followed by Tukey’s multiple comparison test. *P < 0.05, **P < 0.01, ***P < 0.001, ****P < 0.0001, respectively.

A major clinical concern for chemotherapy-treated osteosarcoma patients is metastasis, which is the leading cause of death of the patients. We interrogated this issue by examining the pharmacological effects of SGN1 on the growth and metastasis of osteosarcoma in two different metastatic models. An in situ osteosarcoma model was established by orthotopically injecting MNNG-HOS cells into the proximal tibia of *Balb/c nu/nu* mice. On day 3 post tumor establishment, the mice were randomly divided into three groups and intravenously injected only once with 2 × 10^5^CFU of SGN1per mouse, VNP-V, or PBS at the same volume (Fig. 3A). At the end of treatment (day 12 for the controls and day 25 for the SGN1-treated), SGN1 significantly inhibited the growth of the orthotopic OS compared with both control treatments (Fig. 3B). H&E staining revealed a large number of metastatic pulmonary lesions in both the PBS- and VNP-V-treated groups, whereas no lung metastases were spotted in the SGN1 treated animals (Fig. 3C), even though the lung tissues from the SGN1 treated were collected 13 days later than those from the controls. The incidence of lung metastasis of PBS, VNP-V, and SGN1 treatment was 100%, 83.3%, and 0%, respectively (Fig. 3G). Special AT-rich sequence-binding protein 2 (SATB2) is commonly expressed in osteosarcoma tissues and widely recognized as a definitive marker in the pathological diagnosis of osteosarcoma cell nature [29]. The positive immunohistochemistry staining of SATB2 validated the cell type of the metastatic lesions within the lungs of the PBS- or VNP-V- controls (Fig. S3B). These results indicated that SGN1 has a negative impact on osteosarcoma metastasis.

In a separate system, we constructed a caudal vein metastatic model of osteosarcoma cells in the nude mice (Fig. 3D). In particular, the nude mice at 6 weeks of age were injected with MNNG-HOS cells into the tail veins, and 3 days later, received two intravenous injections (2 weeks apart) of PBS, VNP-V (2 × 10^5^CFU/mouse), or SGN1 (2 × 10^5^CFU/mouse). SGN1 treatment increased median survival by 33.5 days and significantly increased overall survival in comparison to both controls (Fig. 3E). Neither PBS- nor VNP-V treatment was able to control the massive metastasis observed in the liver, lung, and lumbar vertebra (Fig. S3D). H&E staining further confirmed that hepatic, lung, and lumbar vertebra metastases occurred in both the PBS and VNP-V group, while no observable metastases were found in the SGN1 group (Fig. 3F). In total, 7 of 13 mice in PBS-group (53.8%) and 5 of 13 (38.4%) VNP-V treated mice showed massive distal metastatic lesions, whereas none of the SGN1 group displayed any sign of metastasis (Fig. 3G). During the treatment, there were no significant changes in body weight for all groups (Fig. S3A, C). Thus, the test of SGN1 in either of the well-recognized metastatic osteosarcoma models unequivocally demonstrated the inhibitory impact on the growth, and more importantly, on the metastasis of human osteosarcoma-derived models.

### Mechanistic understanding of methionine deprivation impact on cancer cell migration and mitochondrial functions

**Fig. 4:**
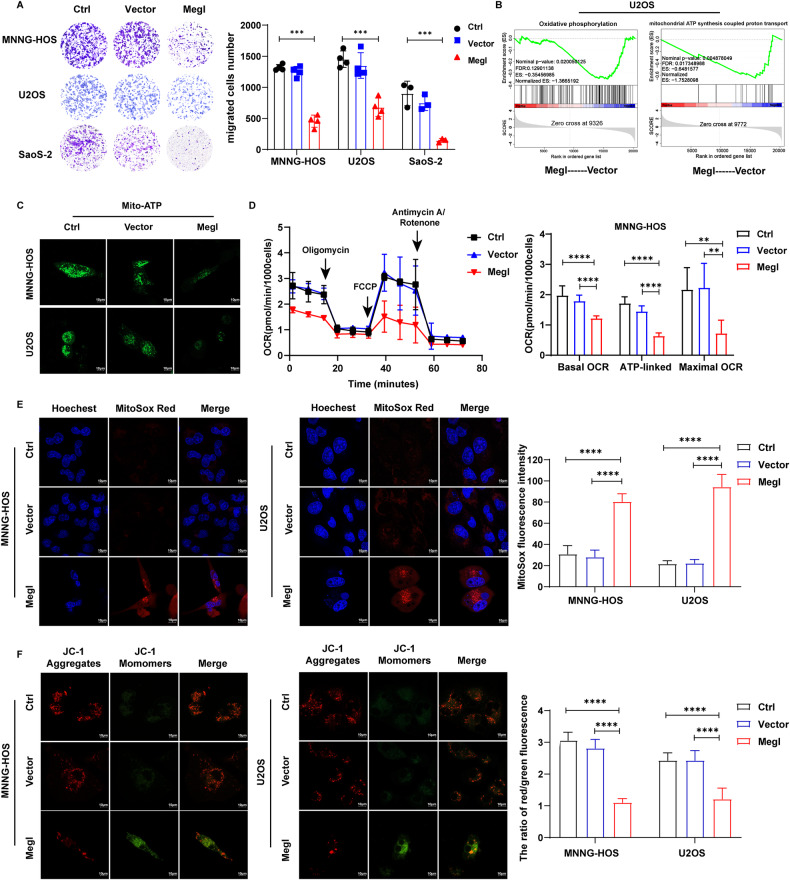
Methionine deprivation promoted apoptosis, inhibited proliferation, migration, and impaired mitochondria function in OS cells. **A** Left panel: transwell image of MNNG-HOS, U2OS, SaoS-2 cells overexpressing *L-methioninase* (Megl); right panel: quantification of transwell assays (*n* = 3). **B** GSEA analysis of oxidative phosphorylation pathway and mitochondrial ATP synthesis-coupled proton transport pathway in *L-methioninase* (Megl) overexpression U2OS cells. **C** The commercial mitochondria ATP probe pCMV-Mito-AT1.03 (mito-ATP) in MNNG-HOS and U2OS cells following *L-methioninase* (Megl) overexpression. Scale bars: 10 µm. **D** Mito Stress Test Kit results of Seahorse cell energy metabolism analysis system in MNNG-HOS cells treated with *L-methioninase* (Megl) expression (*n* = 6). **E** Left panel: the CLSM image of Mito-Sox Red staining of MNNG-HOS and U2OS cells following *L-methioninase* (Megl) overexpression. Scale bars: 10 µm; right panel: quantification of relative Mito-Sox Red fluorescence intensity (*n* = 10). **F** Left panel: the CLSM image of MNNG-HOS and U2OS cells following L-methioninase (Megl) overexpression stained with JC-1. Scale bars: 10 µm; right panel: quantification of relative JC-1 fluorescence intensity (*n* = 10). Data shown as mean ± SD. In (**A**, **D**, **E**, **F**), the *p* values are derived from two-way ANOVA analysis followed by Tukey’s multiple comparison test. The symbols *, **, ***, **** indicate *P* < 0.05, 0.01, 0.001, and 0.0001, respectively.

To solidify the concept that metabolic deprivation can attenuate osteosarcoma metastasis, we transfected the human osteosarcoma cells with the cDNA-encoding *L-methioninase* (Megl) and then performed transwell migration assay. In all three osteosarcoma cell lines, the expression of *L-*methioninase sharply reduced cellular migration (Fig. 4A). Such results clearly resonated with those findings made in the metastatic animal models.

We then assessed the molecular changes induced by the expression of *L-methioninase* in osteosarcoma cells, U2OS, and in a different type of sarcoma, chondrosarcoma cell, SW1353, by RNA-Seq analysis. The inclusion of chondrosarcoma cells allowed us to examine if metabolic deprivation of methionine will lead to similar changes (and by similar mechanisms) in other types of sarcomas as well. Importantly, those genes involved in oxidative phosphorylation pathways (particularly in mitochondrial ATP synthesis-coupled proton transport) were also significantly decreased as evident in the Gene Set Enrichment Analysis (GSEA) (Fig. 4B). Similar findings were also revealed in the chondrosarcoma cells overexpressing the *L-methioninase* (Fig. S4A). Such patterns of gene expression led us to investigate whether methionine deprivation affects mitochondrial function in the osteosarcoma cells. ATP production is one of the hallmarks of mitochondrial strength. The expression of *L-methioninase* significantly decreased the intracellular ATP content in either of the three human sarcoma cell lines (Fig. S4B–D). Moreover, the production of intracellular reactive oxygen species (ROS) was significantly elevated (Fig. S4E–G). We further measured the ATP and ROS in isolated mitochondria. Cultured MNNG-HOS cells and U2OS cells overexpressing the *L-methioninase* were transfected with another construct, pCMV-Mito-AT1.03 (mito-ATP) driving the expression of the AT1.03 protein which allowed us to monitor dynamically ATP concentration changes in the mitochondria [30]. The expression of *L-methioninase* significantly decreased ATP content in OS cells (Fig. 4C), signifying the reduced capacity of ATP production. Moreover, we assessed the oxygen consumption rate (OCR) of cells by Seahorse assay. Overexpression of the *L-methioninase* decreased basal respiration, maximum respiration and ATP production in OS cells (Fig. 4D). Consistent this observation, MitoSOX Red staining of mitochondrial ROS revealed elevated level in the MNNG-HOS and U2OS cells overexpressing the *L-methioninase* compared to that in the controls (Fig. 4E). Importantly, measurements of the JC-1 dye aggregation (in red)/monomer (in green) clearly demonstrated that the expression of *L-methioninase* caused the reduction of membrane potential (Fig. 4F). Quantitative PCR (qPCR) assays confirmed that *L-methioninase* overexpression caused the reduction of the transcriptional level of mitochondrial genome in both osteosarcoma cell lines (Fig. S4H–I). Combined together, these data demonstrated that metabolic deprivation of methionine induced mitochondrial dysfunction in osteosarcoma cells and potentially in other types of sarcomas as well.

### Revelation of the molecular signatures underlying the impact of methionine deprivation on mitochondrial function

**Fig. 5:**
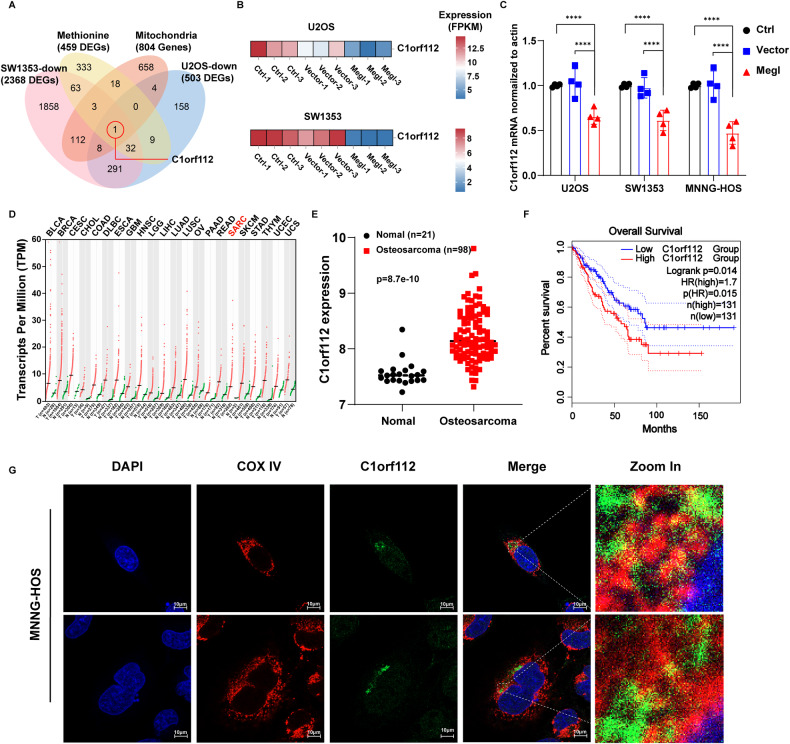
Reduction of *C1orf112* expression in L-methioninase overexpressing osteosarcoma cells. **A** Venn diagram summarizes the number of common genes among the four gene lists: differentially expressed genes from two transcriptome sequencing; 804 human genes mapped to mitochondria from The Human Protein Atlas database (https://www.proteinatlas.org/) and the 459 genes that were significantly positively correlated with the content of methionine in bone tumor cells were analyzed by Pearson method from CCLE database (*R* > 0.5, *P* < 0.05). **B** The heatmap of *C1orf112* expression in the osteosarcoma U2OS and chondrosarcoma SW1353 cells overexpressing the *L-methioninase* (Megl). **C** Quantitative PCR assay to validate the RNA-sequencing results of C1orf112 expression in MNNG-HOS, U2OS, and SW1353 cells following *L-methioninase* (Megl) overexpression (*n* = 4). **D** The expression status of *C1orf112* in different tumor types was visualized by GEPIA 2(GEPIA (Gene Expression Profiling Interactive Analysis) (cancer-pku.cn)). **E** *C1orf112* expression in osteosarcoma tissues compared with normal bone tissue from GSE16088 and GSE33383. **F** Kaplan–Meier overall survival curve of patients divided into high and low-expression of *C1orf112* from TCGA Sarcoma cohort. **G** The image of mitochondrial localization of C1orf112 (Green) in MNNG-HOS cells by utilizing COX IV as a mitochondrial marker (Red) and DAPI (blue) to label the nucleus. Scale bars: 10 µm. In (**C**), the *p* values are derived from two-way ANOVA analysis followed by Tukey’s multiple comparison test. The symbols *, **, ***, **** indicate *P* < 0.05, 0.01, 0.001, and 0.0001, respectively.

The mitochondrial dysfunction induced by methionine deprivation made us to explore what protein(s) plays critical roles in controlling mitochondrial function in the context of cellular methionine restriction. To address this issue, we first performed bioinformatic analysis by examining the matching datasets of transcriptome and metabolome derived from 15 bone tumor cell lines in the Cancer Cell Line Encyclopedia (CCLE). Owing to the small number of human osteosarcoma cell lines collected in this database, the inclusion of 15 bone tumor cell lines was intended for a statistically meaningful and broad understanding of methionine-regulated gene expression. Pearson algorithm identified those genes whose expression levels are statistically correlated with cellular methionine content. Further analysis revealed 459 genes whose expression levels are linearly and positively associated with methionine level (Fig. 5A), and of these 459 genes, only 22 (1 + 3 + 18) encoded proteins are localized to mitochondria, accounting for a small portion of the 804 mitochondrion-localized proteins based on the Human Protein Atlas database (https://www.proteinatlas.org/) (Fig. 5A).

By overlapping the transcriptomic data derived from osteosarcoma U2OS and chondrosarcoma SW1353 cells overexpressing the *L-methioninase* (i.e., methionine-deprived cellular environment), we found 332 genes (1 + 8 + 32 + 291) whose expression was downregulated (Fig. 5A), among which C*1orf112* (Chromosome 1 open reading frame 112) was the only gene whose expression was positively correlated with cellular content of methionine and localized to mitochondria in all 15 bone tumor cells from CCLE database (*R* > 0.5, *p* < 0.05) (Fig. 5A, B). *C1orf112*, a highly conserved protein across various species [31], has been traditionally linked to DNA repair and cell cycle regulation, and proposed to be an indispensable factor in maintaining genome stability [32]. A qPCR and Western blot assay were performed in three different sarcoma cell lines to verify the decreased expression of *C1orf112* as a result of *L-methioninase* expression (Fig. 5C, S5A). Interestingly, the positive relationship between *C1orf112* expression and cellular methionine levels can be extended far beyond bone tumors. Similar to the conclusions drawn from previous reports [33], in-depth analyses of TCGA data revealed at least 22 other tumor types that displayed higher *C1orf112* expression in the lesion tissue than the corresponding normal tissues (Fig. 5D), and such differential expression profile also existed in osteosarcoma samples collected in the Gene Expression Omnibus database (GSE16088 and GSE33383) (Fig. 5E). Importantly, elevated *C1orf112* expression bears a strong clinical significance since it is significantly correlated with poor prognosis of sarcoma patients (Fig. 5F). We further confirmed the mitochondrial localization of C1orf112 (Green) in MNNG-HOS cells by utilizing COX IV (cytochrome c oxidase IV) as a mitochondrial marker (Red) and DAPI to label the nucleus. Our findings revealed that C1orf112 exhibited very limited expression within the nucleus, with the majority being localized at or within the mitochondria (Fig. 5G).

**Fig. 6:**
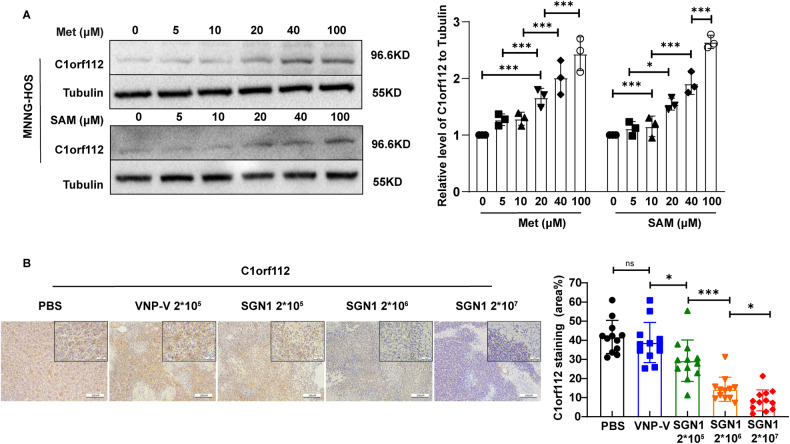
The relationship between the expression of C1orf112 and methionine. **A** Western Blot analysis of C1orf112 expression in MNNG-HOS cells cultured in the medium supplemented with different concentration of methionine or SAM. **B** Left panel: the image of immunohistochemical microphotographs of C1orf112 stained in tumor treated with different doses SGN1. Scale bars: 200 µm/50 µm; right panel: quantitative analysis of C1orf112 positive cells. Data shown as mean ± SD. In (**A**), the *p* values are derived from two-way ANOVA analysis followed by Tukey’s multiple comparison test. In (**B**), the *p* values are derived from one-way ANOVA analysis followed by Tukey’s multiple comparison test. The symbols *, **, ***, **** indicate *P* < 0.05, 0.01, 0.001, and 0.0001, respectively.

With these bioinformatic findings in hand, we experimentally examined the relationship between the expression of C1orf112 and methionine. In the culture medium where we altered methionine supply stepwise, the expression of C1orf112 was gradually elevated as the concentration of methionine or SAM was increased (Fig. 6A). In contrast, immunohistochemical analysis of the tumor tissues treated with SGN1 showed a dose-dependent decrease of C1orf112 expression (Fig. 6B). Combined together, the bioinformatic and experimental data collectively support the concept that *C1orf112* represents a molecular signature in the context of mitochondrial functions, tumor treatment, and methionine deprivation by SGN1.

### The critical role of C1orf112 in mediating methionine deprivation-induced inhibition on the growth and migration of osteosarcoma cells

**Fig. 7:**
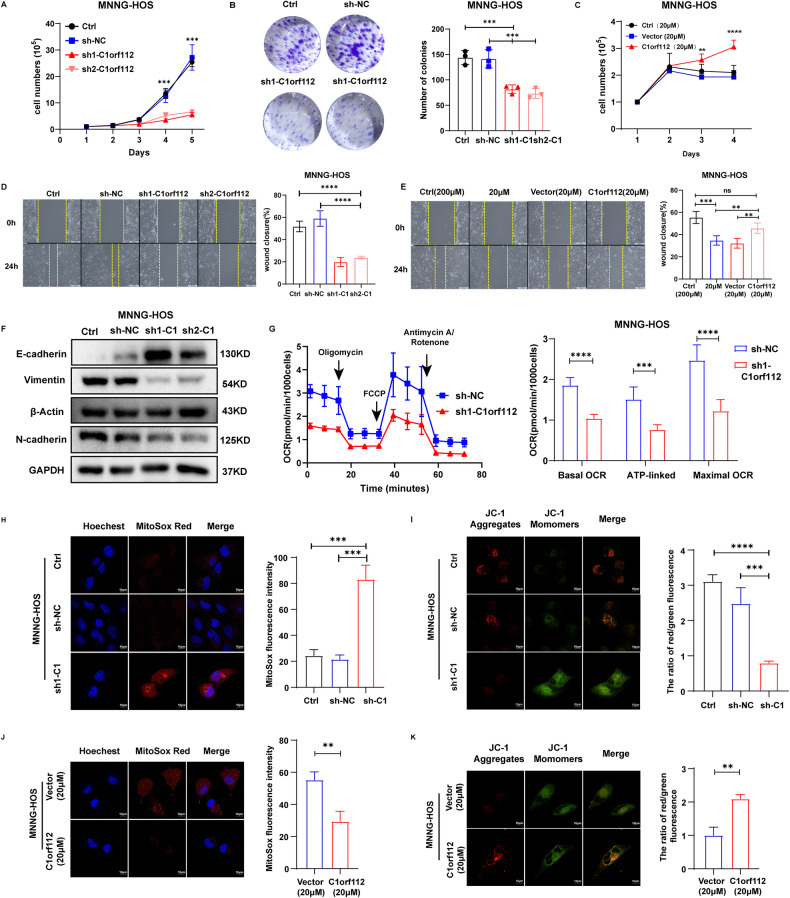
The critical role of *C1orf112* in mediating methionine deprivation-induced inhibition on the growth and migration of osteosarcoma cells. **A** Cell counting assay revealed that silenced *C1orf112* reduced the cell proliferation ability in osteosarcoma (*n* = 3). **B** Left panel: representative images of colony formation assays of the *C1orf112* knockdown MNNG-HOS; right panel: quantification of colony formation assay revealed a reduced rate of cell colony formation after *C1orf112* knockdown (*n* = 3). **C** C1orf112 overexpression promotes MNNG-HOS cell proliferation in the presence of low methionine concentration (20 μM). **D** Left panel: wound healing assay to compare the migratory capabilities of the MNNG-HOS cells with *C1orf112* knockdown; right panel: the quantification of cell migration distance (*n* = 3). **E** Left panel: wound healing assay were performed on methionine deprivation (20 μM) group and C1orf112 -overexpressed group; right panel: quantification of cell migration distance (*n* = 3). **F** Western Blot analysis of the protein expression levels of E-cadherin, N-cadherin, Vimentin expression in C1orf112-silenced MNNG-HOS cells. **G** Mito Stress Test Kit results of Seahorse cell energy metabolism analysis system in MNNG-HOS cells after C1orf112 knockdown (*n* = 6). **H** Left panel: the CLSM image of MNNG-HOS stained with Mito-Sox Red after C1orf112 knockdown, Scale bars: 10 µm; right panel: quantification of relative Mito-Sox Red fluorescence intensity (*n* = 10). **I** Left panel: the CLSM image of MNNG-HOS stained with JC-1 after C1orf112 knockdown, Scale bars: 10 µm; right panel: quantification of relative JC-1 fluorescence intensity (*n* = 10). **J** Left panel: the CLSM image of MNNG-HOS stained with Mito-Sox Red after overexpressing C1orf112 in the presence of low methionine concentration (20 μM), Scale bars: 10 µm; right panel: quantification of relative Mito-Sox Red fluorescence intensity (*n* = 10). **K** Left panel: the CLSM image of MNNG-HOS stained with JC-1 after overexpressing C1orf112 in the presence of low methionine concentration (20 μM), Scale bars: 10 µm; right panel: quantification of relative JC-1 fluorescence intensity (*n* = 10). Data shown as mean ± SD. In (**B**, **D**, **E**, **H**, **I**), the *p* values are derived from one-way ANOVA analysis followed by Tukey’s multiple comparison test. In (**A**, **C**, **G**), the *p* values are from the two-way ANOVA analysis followed by Tukey’s multiple comparison test. In (**J**, **K**), the *p* values are from two-tailed Student’s *t* test. The symbols *, **, ***, **** indicate *P* < 0.05, 0.01, 0.001, and 0.0001, respectively.

The findings above represent the first attempt to link the expression level and function of C1orf112 to mitochondrial function, the growth, and metastasis of cancer cells. To solidify this concept, we generated stable *C1orf112*-knockdown and *C1orf112-*overexpressing osteosarcoma cell lines (Fig. S5B). In the assay of either cultured cell proliferation or colony formation, the knockdown of *C1orf112* with two independent sh-RNAs in the osteosarcoma cells (MNNG-HOS) led to strong inhibition on cell proliferation even in the presence of high medium concentration of methionine (200 µM) (Fig. 7A, B) and overexpression of *C1orf112* neutralized the inhibitory effect of methionine-restriction on osteosarcoma cell proliferation (Fig. 7C). Attenuation of C1orf112 expression resulted in a significant suppression on cell migration (Fig. 7D) while overexpressing of *C1orf112* largely restored the wound closure (Fig. 7E). Tumor metastasis begins with epithelial-mesenchymal transformation (EMT) with the absence of the epithelial marker, E-cadherin, and elevates expression of the mesenchymal markers as the characteristics of EMT. The knockdown of C1orf112 expression led to an increase in E-cadherin, accompanied by a significant decrease in N-cadherin and Vimentin expression (Fig. 7F), indicating the involvement of C1orf112 in regulating the EMT process. The knockdown of C1orf112 in the osteosarcoma cells (MNNG-HOS) led to strong inhibition on cell basal respiration, maximum respiration, and ATP production (Fig. 7G). Attenuation of *C1orf112* expression also led to an increased Mito-ROS while a drastic suppression on mitochondrial membrane potential (Fig. 7H, I). In contrast, overexpression of *C1orf112* decreased the Mito-ROS (Fig. 7J) while largely restoring the mitochondrial membrane potential (Fig. 7K). Quantitative analysis of mitochondrial gene expression further supported the concept that alterations of *C1orf112* expression levels modulate mitochondrial gene expression profiles, particularly those genes involved oxidative phosphorylation (Fig. S5C, D). Combined together, these data strongly supported the critical functions of *C1orf112* far beyond the traditionally defined role in controlling nuclear DNA replication, particularly in controlling osteosarcoma growth, metastasis, and mitochondrial function in the context of cellular methionine levels.

The influence of methionine-restriction on C1orf112 protein expression was likely achieved at least in part through the recognition of epigenetically modified RNA transcript (m6A). In further analyzing the transcriptomic data involving the readers of m6A-modified mRNA, we found that the expression of *HNRNPA2B1*, a primary reader of m6A [34], displayed the most significant reduction among all analyzed readers (Fig. S5E). In searching the M6A2Target database (http://m6a2target.canceromics.org), *C1orf112* is shown as one of the target gene of *HNRNPA2B1*. Further analysis of TCGA database revealed that the expression of *HNRNPA2B1*is strongly and positively correlated with that of *C1orf112* in sarcoma (Fig. S5F). Thus, methionine restriction can decrease RNA m6A modification as well as the expression of *HNRNPA2B1*, both of which can cause the reduction of C1orf112 protein expression and eventually alter the proliferation and metastatic phenotype of osteosarcoma (and potentially other sarcoma) cells. Collectively, these data strongly suggest that the expression level of *C1orf112*, a newly identified molecular signature of cancer cells in the context of methionine deprivation, plays a pivotal role in mediating methionine-deprivation induced osteosarcoma cell growth and metastasis, and in this context, such effects of *C1orf112* are tightly coupled with the regulation of mitochondrion oxidative phosphorylation activity.

## Discussion

Osteosarcoma often inflicts life-threatening damage in the bone tissue of young people, and the overall survival rate has made little progress over the past 30 years owing to the frequent recurrence that is manifested as pulmonary and other essential organs metastatic lesions [35]. In this study, we have designed a strategy to target the strong methionine dependency of most types of malignancies and demonstrated the strong inhibitory effect of methionine deprivation on osteosarcoma growth and, more importantly, metastasis. The collective findings lay the foundation for developing a novel treatment strategy for osteosarcoma patients.

Methionine is an essential amino acid that is required for such important cellular processes as protein synthesis, DNA synthesis, and epigenetic modifications (such as DNA or histone methylation) [36, 37]. Cancer cells, including tumor-initiating cells, have a much higher dependency on methionine requirement than normal non-cancerous cells due to the deficient enzymatic activity in the “methionine salvage pathway” while facing methionine restriction [38–40]. Recent pharmacological efforts have focused on the development of inhibitors of methionine metabolic pathways, low methionine diets, or recombinant L-methioninase as the tumor-killing strategies in a variety of tumors [41–43]. Each of the strategies has its own caveats in the clinical application. The dietary approach of methionine restriction can induce systemic malnutrition, which is a serious issue for advanced stage cancer patients who already have cachexia [7]. The small molecule inhibitors developed thus far are often met with some unacceptable serious adverse events due to the off-target actions on non-tumor tissues [44]. Although injection of recombinant methioninase holds the promise to systemically deplete methionine, the strong antigenicity with a short circulating half-life presented itself as a non-viable clinical option [45]. More importantly, neither the inhibitors nor the recombinant methioninase proteins have the ability to reduce methionine content, specifically in the tumor tissues.

On this note, the recently developed SGN1, a genetically engineered bacterium drug (currently in global phase I trial), can fulfill this requirement [22, 46]. Bacteria have the natural ability to target tumor tissues with insufficient oxygen and low pH owing to their high motility and chemoreceptors [47]. SGN1 still retains the ability to colonize in experimental tumors and overexpresses the L-methioninase to hydrolyze and deprive methionine (as well as SAM/SAH) only in the osteosarcoma tissues [22]. The treatment with SGN1 led to drastic retardation of osteosarcoma growth and pulmonary (also hepatic) metastasis in the orthotopic and metastatic models, and significantly prolonged the survival of the osteosarcoma metastatic model. We should also add that such therapeutic effects may be extended to other types of sarcomas as well.

Mitochondrial dysfunction induced by methionine restriction plays a key role in the control of osteosarcoma growth and metastasis. Owing to the diminished oxidative phosphorylation capability, many types of cancer cells primarily rely on glycolysis for ATP production [48, 49]. However, a series of recent studies revealed the importance of the oxidative phosphorylation by pharmacologically inhibiting mitochondria function, particularly by targeting oxidative phosphorylation pathway, in controlling the growth of metastatic tumor cells [50], therapy-resistant tumor cells [51] and cancer stem cells [52], thus linking the mitochondrial function with cancer cells’ ability to metastasize. Indeed, our transcriptomic analysis revealed a significant reduction in the expression of those genes involved in oxidative phosphorylation and TCA cycle as a result of L-methioninase overexpression and methionine reduction in the osteosarcoma cells. In addition, methionine deprivation either in culture medium or through L-methioninase overexpression induced mitochondrial dysfunction in osteosarcoma cells, as evident in their elevated Mito-ROS production, reduced ATP production, decreased basal and maximum respiration, and damaged mitochondrial membrane potential. These results are also consistent with earlier published findings that methionine restriction destroys mitochondrial functions in breast cancer cells [53].

Transcriptomic and bioinformatic analyses allowed us to further zero in on the reduction of *C1orf112* protein expression induced by methionine deprivation as the key event in the context of mitochondrial dysfunctions and growth/metastasis of osteosarcoma. Though not extensively characterized, *C1orf112* is traditionally defined as a nuclear protein involved in the regulation of DNA replication and DNA damage responses [31, 32]. Previous research [33] and our bioinformatic analysis discovered that high *C1orf112* expression significantly correlated with reduced progression-free survival and overall survival in a wide variety of cancer types, particularly sarcoma patients. This study represents the first to show that the cellular functions of *C1orf112* should be extended far beyond the realm of nuclear DNA replication, and that this protein can influence mitochondria, and consequently the growth and metastasis of osteosarcoma. Accordingly, C1orf112 protein expression is positively correlated with methionine content in osteosarcoma cells, further implying that C1orf112 expression is regulated by methionine supply. We further confirmed that C1orf112 exhibited very limited expression within the nucleus, with the majority being localized at or within the mitochondria. The knockdown of *C1orf112* expression negatively affected the growth and migration of osteosarcoma cells, and sharply decreased their mitochondrial functions even in the culture medium with high concentrations of methionine. In contrast, overexpression of *C1orf112* largely restored the mitochondrial function, cell growth, and cellular migration of osteosarcoma caused by the deprivation of methionine. These results provided strong support for the concept of C1orf112 regulating mitochondrial function in osteosarcoma cells. Although the mechanisms underlying methionine-regulated *C1orf112* expression deserve further investigation, the epigenetic modification of the *C1orf112* mRNA (m6A) is likely a primary pathway. Already, *C1orf112* mRNA is a target gene of m6A reader, *HNRNPA2B1*, and methionine restriction can lead to a dramatic reduction in m6A signals in human lung cancer cells (data not shown). These findings, coupled with the drop of SAM, the provider of methyl group to the methylation of DNA, RNA, and histones [54–56], should help us precisely define the mechanisms about how methionine level influences the expression of *C1orf112*, which in turn controls the mitochondrial functions, the growth, and metastasis of osteosarcoma.

## Conclusions

Taken together, our data revealed a potential novel treatment strategy that can strongly stall the growth, and particularly metastasis of osteosarcoma, as evident in several demonstrated animal models. This strategy entails targeting the unusually high requirement of methionine of osteosarcoma cells through a genetically engineered *Salmonella* strain to deliver the expression of *L-methioninase* and to specifically deprive methionine in the malignant tissues, thereby slowing tumor development and metastasis. We have further revealed for the first time that methionine deficiency can induce impairment of mitochondrial functions in osteosarcoma cells and a primary molecule event underlying this impact is the expression reduction of C1orf112 localized to mitochondria. Given the potent effect of tumor-targeted methionine deprivation on malignant growth and metastasis, the findings shed light on a novel treatment modality in not only osteosarcoma but other types of sarcomas as well.

## Material and methods

### Cell culture

Osteosarcoma cell lines MNNG-HOS, U2OS, Saos-2 (these cell lines were chosen for this study due to their high degree of malignancy, tumorigenicity, characteristics of cancer stem cells (CSCs), in vitro sphere formation capability, and drug resistance [57–59]) and chondrosarcoma cell SW1353 were purchased from the Shanghai Institute of Cell Biology (Chinese Academy of Medical Sciences, Shanghai, China). MNNG-HOS, U2OS, Saos-2 and SW1353 cells were cultured, respectively, in MEM (Gibco, 41500034), McCOY′s 5A (Sigma, M4892, U2OS and Saos-2) and DMEM (Gibco, 12800017) medium containing 10–15% FBS (RNC RC101-003) at 37 °C in a humidified 5% CO_2_ incubator. All cells were identified by short tandem repeat (STR) profiling and regularly tested for mycoplasma contamination. For methionine or SAM restriction protocol, cells were cultured in methionine-free medium (Gibco,21013024) and supplemented with different concentrations of methionine (Sigma M5308-25G) or SAM (Sigma 3493-13-8). In the case of methionine-dependency test, MNNG-HOS, U2OS, Saos-2 cells were cultured in MET−homocysteine+ (HCY, 100 μM, Sigma A9384-10MG) or MET + (MET, 200 μM, Sigma M5308-25G) HCY−medium.

### Bacterial strains

S. Typhimurium strain SGN1 and VNP20009-V(VNP-V) were generously provided by Guangzhou Sinogen Pharmaceutical Co., Ltd, Guangzhou, Guangdong Province, China [22]. SGN1 and VNP20009-V bacteria were cultured overnight in Luria-Bertani (LB) broth from a single colony, subcultured (1:50) to mid-logarithmic phase the next day, and adjusted to an appropriate concentration in normal saline based on an optical density reading at 600 nm and the flat colony counting method.

### Animals

The 4- to 6- weeks-old female mice (*BALB/C-nu/nu*, NOD-SCID) were purchased from the GemPharmatech. All experiments were conducted with protocols approved by Laboratory Animal Center of Guang Dong Pharmaceutical University (Guangzhou, PR China). Animal care procedures strictly follow the animal care guidelines formulated by the animal care committee of Guangdong Pharmaceutical University (Guangzhou, PR China, Ethics permit number: gdpulacspf2017064). For subcutaneous xenografts, single MNNG-HOS cells (5 × 10^6^/mice) were mixed with serum-free MEM and subcutaneously injected into the lateral body of nude mice. Tumor volume was calculated by the formula 0.52 × *l* × *w*^2^, where *l* and *w* are tumor length and width, respectively. For the situ model, a lateral incision was made on the lower femur or an anterolateral incision on the tibial trochanter. The subcutaneous tissue was then cut to expose either the lower femur or metaphyseal region of the upper tibia and the 1 × 10^7^ cells suspension was injection into the right tibial medullary cavity of nude mice. The tumor volume of orthotopic OS were measured in the anteroposterior (AP) and lateral (L) planes 3–4 times weekly using digital caliper and the volume was calculated using 4/3π [1/4(AP + L)]^2^ [60]. For PDX model, the clinical osteosarcoma samples used in the current study were collected from the Department of Orthopaedic Oncology, Guangdong People’s Hospital, Guangzhou, China, and obtained written informed consent from patients and/or their immediate family members. In this study, we obtained fresh tissue biopsies from the patients and constructed the PDX models. In this process, we also confirmed the pathology from the hospital. The immunohistochemistry staining revealed CDK4(+), P16(-), P53(-), SATB2(+++), Cyclin D1(+), Ki67(++), confirming the diagnosis of osteosarcoma. All PDX tumors were maintained by passage to new NOD-SCID receptor mice without cell culture. As previously described [61], the PDX tumor was isolated and injected. In simple terms, tumors were collected in cold PBS with antibiotics, cut into small pieces about 2 mm in diameter with a sterile blade, and implanted into the side of NOD-SCID mice with an implant needle. The mice were randomly assigned. Tumor volume was calculated by the formula 0.52 × *l* × *w*^2^, where *l* and *w* are tumor length and width, respectively. In accordance with the Laboratory Animal Care Regulations, the mice were euthanized in all experiments once the tumor volume reached 1500–2000 mm^3^, and the tumor was collected for analysis.

### Organoids culture and treatment

This experiment followed a published protocol for generating human osteosarcoma with some modifications [62]. For PDO model, the clinical osteosarcoma samples used in the current study were collected from the Department of Orthopaedic Oncology, Guangdong People’s Hospital, Guangzhou, China, and obtained written informed consent from patients and/or their immediate family members. The tumor samples were minced into small pellets on ice after washing with PBS. The minced tissues were incubated with 1 mL TrypLE (Gibco, A1217701) at 37 °C for 45 min and washed with DMEM containing penicillin, streptomycin (Gibco,15140122) and nystatin (Sigma N9150-20 mL). After digestion, the cell suspension was filtered through a 70 μm cell strainer to remove undigested large debris and centrifuged at an average of 300 rpm for 5 min to pellet cells. The isolated cells were re-suspended in a complete culture media and mixed with Matrigel matrix at a ratio of 1:2 for 3D culture in the form of about 3000 cells/30 μL droplet/well. After the matrigel had completely solidified, a complete medium was added and the organoid was culture at 37 °C and changed every 2 days. The complete culture medium for osteosarcoma organoids refers to earlier studies [63]. For drug sensitivity assay, organoids were treated with l μM doxorubicin/DOX (Med-CheExpress, MCE, HY-15142) [64], 4 μM cisplatin/DDP (MCE,HY17394) [27], 10 μM methotrexate/MTX (MCE, HY-14519) [65], or 10 μg/mL (MCE, HY-17419) ifosfamide/IFO [65], 10 μg/ml pembrolizumab (Selleck, A2005) [66] or equivalent DMSO vehicle for 96 h. For bacteria treatment, isolated cells were re-suspended in a complete culture media and mixed with Matrigel matrix and bacteria at a ratio of 1:2:1 for 3D culture in the form of about 3000 cells/30 μL droplet/well. SGN1 and VNP20009-V bacteria (5 bacteria for every cell) were co-imbedded with organoid in matrigel for 96 h. After 72 h, the spheroids were incubated overnight in a medium containing 10 μM EdU (Beyotime Biotech C0078S) permeable red fluorescent dye for cell proliferation test. TUNEL cell apoptosis detection kit (Beyotime C1088) was used to detect the apoptotic cells in the spheroids by the methods described elsewhere. For recovering organoids from Matrigel matrix, the culture can be treated with Cell Recovery Solution (Corning, 354253) to release organoids and then proceed with fixing, permeabilizing, and staining the organoids [67]. The immunofluorescence images were collected in 20X magnification, the number of EdU (+) and TUNEL (+) cells was measured in 15 organoids with diameters 60–100 μm (the organoids cultured within this range typically exhibit good viability) in each group. The organoids images were further enlarged to the appropriate sizes and calculated the percentage of EDU and TUNEL marker-positive cells out of the total number of Hochest+ cells with ImageJ software. The representative image in the text is sourced from one of the patients, and the statistics included the data from two patients. The representative image of PDOs is sourced from one of the patients, and the statistics included the data from two patients.

### Metabolite extraction and quantitation

Prior to LC-MS/MS analysis, osteosarcoma cells (1 × 10^6^ cells) and tissue (5–10 mg) were thoroughly mixed or homogenized (tissue) in 1 mL of buffer containing 60% methanol and 40% ddH2O, which was pre-cooled to −40 °C. The mixture was then centrifuged. Amino acids were the UltiMate 3000-TSQ Endura UPLC–MS/MS system from Thermo Fisher Scientific, equipped with a Syncronis HILIC Column (100 × 2.1 mm, 1.7 μm). Mass spectrometric detection was performed on a TSQ Endura triple quadruple mass spectrometer with an elect ionization source. Compound-specific parameters of the mass spectrometer were set as follows: spray voltage at 3500 V, capillary temperature at 320 °C, vaporizer temperature at 350 °C, sheath gas at 35 (Arb), and auxiliary gas at (10b). The detections were carried out in the (SRM) positive mode with transitions of m/z values as follows: Met - m/z150.2 → 104.1511; SAH - m/z385.45 → 134.111; SAM - m/z399.35 → 250.111 respectively. Instrument control and data acquisition were performed using an Xcalibur workstation from Thermo Fisher Scientific.

### Histopathology and immunohistochemistry

Tissues were fixed in 4% buffered formalin (ServicebioG1101-25L) and embedded in paraffin. For histopathological visualization, 4 μm tissue sections were stained with H&E. Antibodies used for immunostaining included those against the following: anti-C1orf112 (1:100 Thermofisher, PA5-55082,), anti-Ki-67 (1:200 CST 12202S), anti-Cleaved-caspase3 (1:400 CST #9661), anti-SATB2 (1:600 CST #39229) as previous described.

### Bioinformatics analysis

The genes whose protein products have evidence of location in mitochondria were downloaded by the Human Protein Atlas database (https://www.proteinatlas.org/). The GEO dataset GSE16088 and GSE33383 were downloaded and analyzed by R (version 4.2.1, https://www.r-project.org/). Characteristics of GEO datasets are in Supplementary Table 1. The matching datasets of transcriptome and metabolome derived from 15 bone tumor cell lines were downloaded by the CCLE dataset (https://portals.broadinstitute.org/ccle). The different expressed genes were calculated with R v4.0.3 software package ggplot2 (v3.3.3) and identified by the threshold criteria of log2 Fold-change (FC) ≥ 1.00 and *p* < 0.05. GO and KEGG analysis was performed to investigate the potential role of genes.

### Co-culture of dosages bacteria and cancer cells

Cells were co-cultured with the bacteria of different genotypes (Multiplicity of infection was 1:5; 1:10; 1:20; 1:30). After adding the bacteria, the plates were centrifuged for 5 min at 600 × *g* before being incubated for 3 h at 37 °C. Extracellular bacteria were washed with gentamicin-containing PBS and continued to be cultured in a gentamicin-containing medium until 24 h.

### Plasmids and stable-cell line establishment

Small hairpin RNA (shRNA) was obtained from Kidan Biosciences (Shanghai, China). The gene sequences of shRNA were listed as follows:

sh*C1orf112*-1F: CCGGAGACCACTCTAAGGAATATTTCTCGAGAAATATTCCTTAGAGTGGTCTTTTTTG;

sh*C1orf112*-2F: CCGGGCTTCCTGACTATGTTCGTTTCTCGAGAAACGAACATAGTCAGGAAGCTTTTTG;

sh*C1orf112*-3F: CCGGGTCACCTTGTATCAGCATGTTCTCGAGAACATGCTGATACAAGGTGACTTTTTG. For stable *C1orf112* knocking down cell line construction, shRNAs was cloned into the PLKO-U6-GFP-PURO (Kidan Biosciences, China). pLKO.1 construct together with packaging vectors psPAX2 and pMD2.G (Addgene) were co-transfected into 293 T cells for lentivirus production. For stable *L-methioninase* over-expressed cell line construction, LV-PGK and *L-methioninase* (Cyagen, China) and pcDH-cmv-mcs-ef1-egfp-t2 and *C1orf112* plasmids (Kidan Biosciences, China) were co-transfected with lentiviral packaging vectors psPAX2 and pMD2.G into 293 T cells. Viruses were harvested at 48 h and 72 h for cancer cell infection. Then, stable cell lines were selected by puromycin (2 μg/mL) for 1 week.

### Cell Proliferation, Apoptosis and Migration assays

For cell proliferation assay, ~1 × 10^5^ cells were plated in 6-well plates and counted by cell counting at 24 h, 48 h, 72 h, and 96 h. For assessing cell death assay, the final concentrations of the apoptosis inhibitor Z-VAD-FMK and necrosis inhibitor Necrostatin-1 were 10 μM and 50 μM, respectively, in a 24-well plate containing 5 × 10^4^ cells per well. The cell count was determined after a 48-h incubation period. For apoptosis assay, we conducted experiments at various time points (1 h, 3 h, 5 h, and 8 h) and observed that SGN1 exhibited a heightened apoptotic induction effect without causing excessive bacterial proliferation or changing the pH of the medium, and the treatment time was determined as 5 h. The Annexin V-PE binding capability of the treated cells (30 bacteria for every cell/ *L-methioninase* overexpressing cell line) was evaluated by flow cytometry using the Annexin V-PE/7-amino-actinomycin Apoptosis Detection Kit (BD 559763) to detect apoptosis. Cell migration assay for *L-methioninase* overexpressing cell line was performed by using 24-well Millicell (Sigma-Alorich CLS3422). 1 × 10^4^ cells per well were plated on transwell chambers. A complete medium containing 20% FBS served as a chemoattractant in the lower chamber. After 24 h, the invading cells were fixed with 4% para-formaldehyde and stained with 0.1% crystal violet. Randomly selected fields were taken for quantifying the migrating cells. A scratch wound assay was also used to determine cell migration. Cells were plated at a concentration of 5 × 10^5^ cells per well in triplicate into 6 well plates and cultured for 24 h to reach 80% confluency, and then a straight artificial wound was scraped with a 200 μl pipette tip. The cell migration ability was measured by photographing the distance at 0 and 24 h. Wound closure rate (%) = (area of initial scratch − the area of final imaged cell-free area)/area of initial scratch × 100.

### Colony formation assay

The *L-methioninase* overexpressing cells were seeded at a density of 500 per well in 6-well plates and incubated for 10–14 days. Subsequently, the cells were rinsed with PBS, fixed in 4% paraformaldehyde for 15 min, and stained with 2% crystal violet solution for 15 min at room temperature. The colonies consisting of 50 or more cells were manually counted.

### Mitochondrial membrane potential (MMP) assay and ROS detection

The JC-1 Assay Kit (Beyotime) was used to assess MMP in osteosarcoma cells. The stably transfected L-methionase osteosarcoma cells were seeded into 24-well plates and confocal dishes at a density of 5 × 10^4^/well and 1 × 10^3^/well for a duration of 48 h. The JC-1 probe was introduced to cell media and incubated at 37 °C for 20 min, and then observed under fluorescence microscopy (Olympus) and a confocal laser scanning microscopy (CLSM) with the emissions wavelength at 525 nm for green fluorescence detection and at 590 nm for red fluorescence. For quantification of intracellular ROS and Mito-ROS, osteosarcoma cells were plated at a concentration of 1 × 10^5^/well and 1 × 10^3^/well in 6-well plates and confocal dishes for a duration of 48 h. Cells were incubated with 10 μM DCFH-DA (Beyotime) and 0.1 μM MitoSox Red (Thermo Fisher) at 37 °C in the dark for 20 min and observed under a flow cytometry (DxP Athena Cytomter) and confocal laser scanning microscopy (CLSM, LSM 800 With Airscan). Fluorescence intensity was performed on the figures by using the ImageJ software.

### Measurement of ATP

Intracellular ATP concentrations were determined using an ATP assay kit (Beyotime, China), according to the manufacturer’s instructions. Cells at 70–80% confluency were resuspended to 2 × 10^6^ cells/well in 6-well plates and 0.2 ml lysate to lyse the cells. To measure luminescence, a multimode microplate reader (Thermo Scientific Varioskan™ LUX) was employed. pCMV-Mito-AT1.03 (mito-ATP, Beyotime, China) is a commercial dye for mitochondrial ATP, which is a CMV promoter used to express AT1.03 protein with mitochondrial localization signal (MLS) in mammalian mitochondria, suitable for real-time monitoring of ATP concentration changes in the mitochondria [30]. According to the manufacturer’s instructions, osteosarcoma cells were plated at a concentration of 1 × 10^3^/well in confocal dishes and transfected with pCMV-Mito-AT1.03 plasmids using the Lipofectamine 3000 Transfection Reagent (ThermoFisher, USA) for a duration of 48 h and observed under a confocal laser scanning microscopy (CLSM, LSM 800 With Airscan).

### Seahorse assay

The cells were inoculated into XFe96 microplates (Seahorse Bioscience). oxygen consumption (OCR) readings were obtained under basal conditions and after the addition of mitochondrial inhibitors (oligomycin (1.5 μM), carbonyl cyanide-4-(trifluoromethoxy) phenylhydrazone (FCCP, 1.0 μM) and rotenone/antimycin A (0.5 μM)). Coupling efficiency, which reflects the proportion of respiratory activity utilized for ATP production, was determined by calculating the percentage change in OCR immediately following oligomycin treatment compared to the final baseline value. Hippocampal data was normalized to cell counts using DAPI fluorescent dye signals immediately after hippocampal analysis.

### RNA sequencing analysis and qRT-PCR

Total RNA was extracted from cell tissues using a TRIzol reagent kit (Invitrogen). PCR was amplified and sequenced using Illumina NovaSeq 6000 by Gene Denovo Biotechnology (Guangzhou, China). We identified genes as significantly differentially expressed if they had a fold change ≥1 and a false discovery rate <0.05. Total RNA was isolated from osteosarcoma cell lines using the Total RNA Kit (R6834-01, Omega). One microgram of RNA was used for cDNA synthesis using a High-Capacity cDNA Reverse Transcription Kit (Q711-02, Vazyme). Transcript levels were determined on an Applied Biosystems QuantStudio 5 real-time PCR system using the Vazyme iTaq Universal SYBR Green Supermix (R223-01, Vazyme). Relative gene expression was calculated using the 2 -∆∆Ct method. The primer sequences are listed in Supplementary Table 2.

### Western blotting

Protein extraction was performed in protein lysis buffer (50 mM Tris-HCl pH 7.5, 150 mM NaCl, 0.5% NP-40) supplemented with 50X protease and phosphatase inhibitor cocktail for general use (Beyotime P1046). A total of 30 μg of protein extracts was separated on 10% SDS-PAGE, transferred to a PVDF membrane (Millipore), and blotted with antibodies against the following: anti-C1orf112 (Thermofisher, PA5-55082,), anti-Tubulin (CST-2125S), anti-beta-Actin (Affinity-T0022), anti-GAPDH (Affinity-AF7021), anti-E-cadherin (Affinity-BF0219), anti-N-cadherin (Affinity-AF5239), anti-Vimentin (Affinity-BF8006), anti-Cleaved PARP (CST-9541S), anti-Bcl-2 (SANTACRUZ, sc-7382). Densitometry was performed on the Western blot figures by using the ImageJ software.

### Co-localization of mitochondria and C1orf112

Osteosarcoma cells were plated at a concentration of 1 × 10^3^/well in confocal dishes and stained with anti-COX IV (CST-1967S, 1:200) and anti-C1orf112 (1:100) antibodies, respectively, and DAPI to label the nucleus. Cells were observed under a confocal laser scanning microscopy (CLSM, LSM 800 With Airscan).

### Statistical analysis

The statistical analysis was performed using GraphPad Prism 8 software. *P* ≤ 0.05 was considered significant. For pairwise comparison, we employed the unpaired 2-tailed Mann-Whitney *t* test for 2-group comparisons or one-way ANOVA or two-way ANOVA analysis followed by Tukey’s multiple comparison test for 3 or more groups.

### Supplementary information

SUPPLEMENTARY MATERIAL

## Data Availability

RNA data FASTQ files and processed normalized output have been deposited in the NCBI Sequence Read Archive database (accession number: PRJNA1023390). All raw, uncropped Western blots are available as Supplementary Material. Additional details regarding data and protocols that support the findings of this study are available from the corresponding author upon request.
