# Supplementary material for: Deprivation of methionine inhibits osteosarcoma growth and metastasis via C1orf112-mediated regulation of mitochondrial functions
Source: Cell Death Dis. 2024 May 20;15(5):349. doi: 10.1038/s41419-024-06727-1 (PMC11106329; doi:10.1038/s41419-024-06727-1)

Fig S1

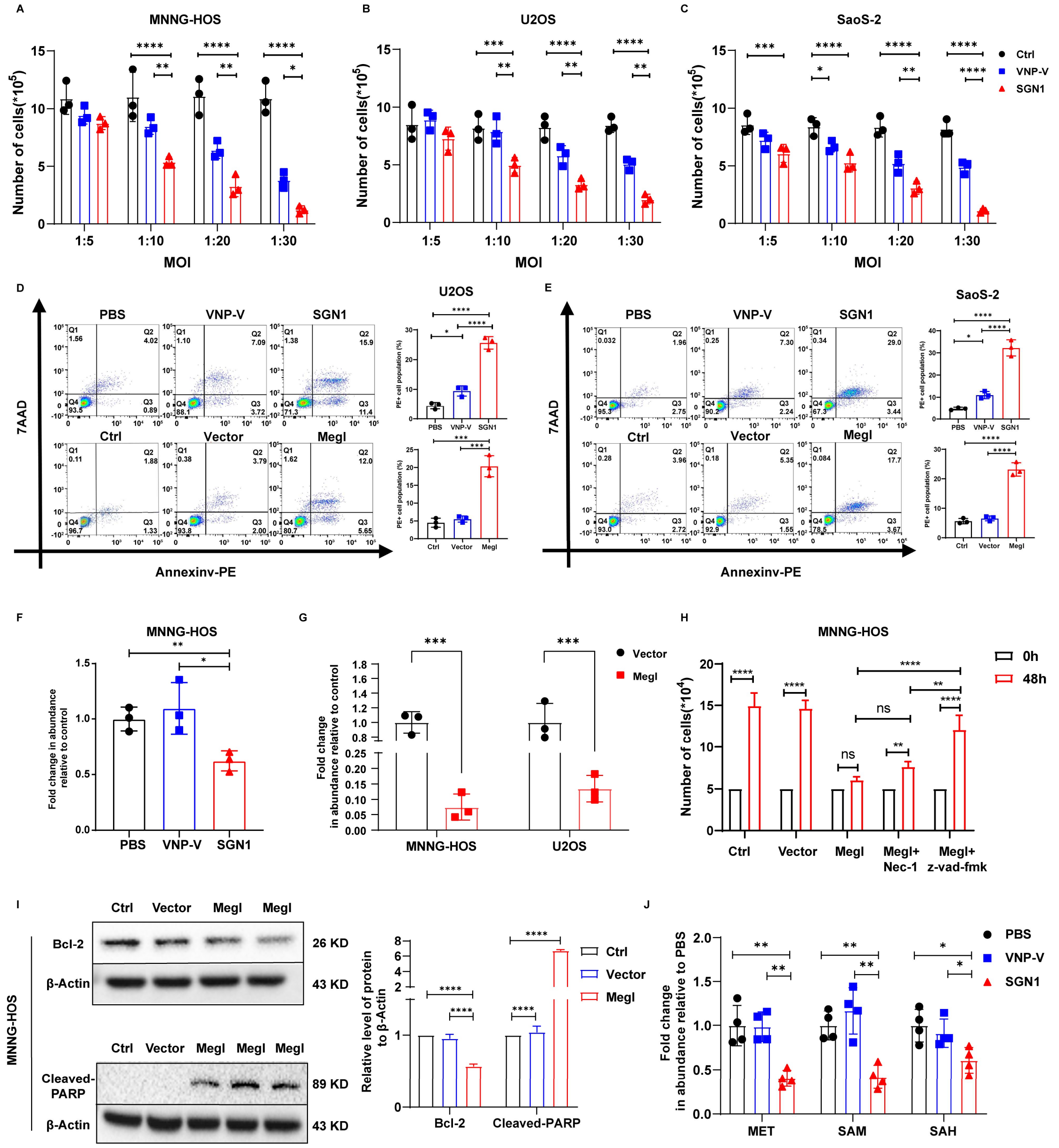

Fig S1. SGN1 generated cytotoxic effects on osteosarcoma cells in a dose-dependent manner. A-C. The effects of osteosarcoma cells proliferation co-cultured with SGN1 by cell counting assay. Osteosarcoma cells were co-cultured with different dose manner of VNP-V and SGN1for 24h. **(D.E).** The apoptosis-inducing effect of different types of bacteria or *L-methioninase* (Megl) was investigated by flow cytometric analysis of U2OS and Saos-2 cells stained with Annexin V and 7-AAD (n = 3). **F.** LC-MS/MS analysis of the methionine after co-culture with SGN1 for 5 hours (n=3) *$p < 0.05$. **G.** LC–MS assay showed decreased methionine levels in osteosarcoma cells (MNNG-HOS and U2OS) overexpressing *L-methioninase* (n=3). **H.** The restore effects of Nec-1 and z-vad-fmk to MNNG-HOS overexpressing *L-methioninase* by cell counting assay (n=3). **I.** Western Blot

analysis of the protein expression levels of Bcl-2, Cleaved-PARP expression in overexpressing *L-methioninase* MNNG-HOS cells**. J.** LC-MS/MS analysis of the intratumoural methionine (MET), SAM, and SAH after the treatment of vehicle (PBS), VNP-V (2×10^6 CFU/ mouse), SGN1(2×10^6 CFU/ mouse), with values normalized to abundance in the PBS condition. Data shown as mean ± SD. In D-F, the p values are derived from one-way ANOVA analysis followed by Tukey's multiple comparison test. In A-C, G-J, the p values are derived from two-way ANOVA analysis followed by Tukey's multiple comparison test. The symbols *, **, ***, ****indicate $p < 0.05$, 0.01, 0.001, and 0.0001, respectively.

Fig S2

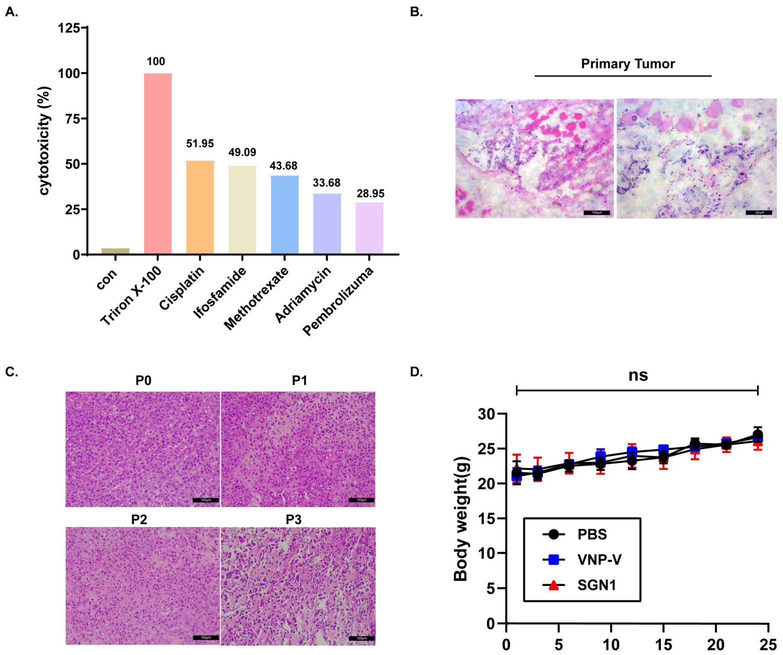

Fig S2. Osteosarcoma PDO model and PDX model was generated successfully

A. Sensitivity of Patient-Derived Organoids (PDOs) to the first-line chemotherapy drugs. **B**. Representative microphotographs of H&E staining for primary tumor from patient and mice**(C)** carrying third-generation tumors （n=4). **D.** The body weight of mice in osteosarcoma PDX models after treatment with SGN1. The *p* values are from two-way ANOVA analysis followed by Tukey's multiple comparison test. and the symbols **ns** indicate $p > 0.05$.

Fig S3

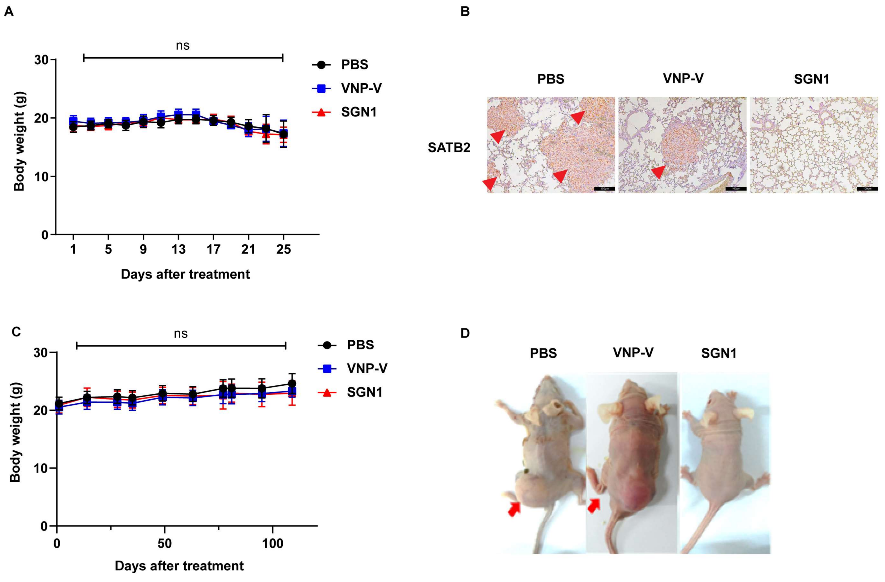

Fig S3. SGN1 inhibited the distant metastasis of osteosarcoma in *vivo*.

A. The body weight of mice in MNNG-HOS orthotopic model after SGN1 treatment. **B.** Representative microphotographs of immunofluorescence staining for SATB2 of lung metastases(triangle) from situ Osteosarcoma model. Scale bars:100 μm. **C**. The body weight of mice with MNNG-HOS caudal vein metastasis model after treatment with SGN1. **D.** The image of lumbar vertebra metastases (triangle) mice. The p values are from two-way ANOVA analysis followed by Tukey's multiple comparison test. and the symbols ns indicate $p > 0.05$.

Fig S4

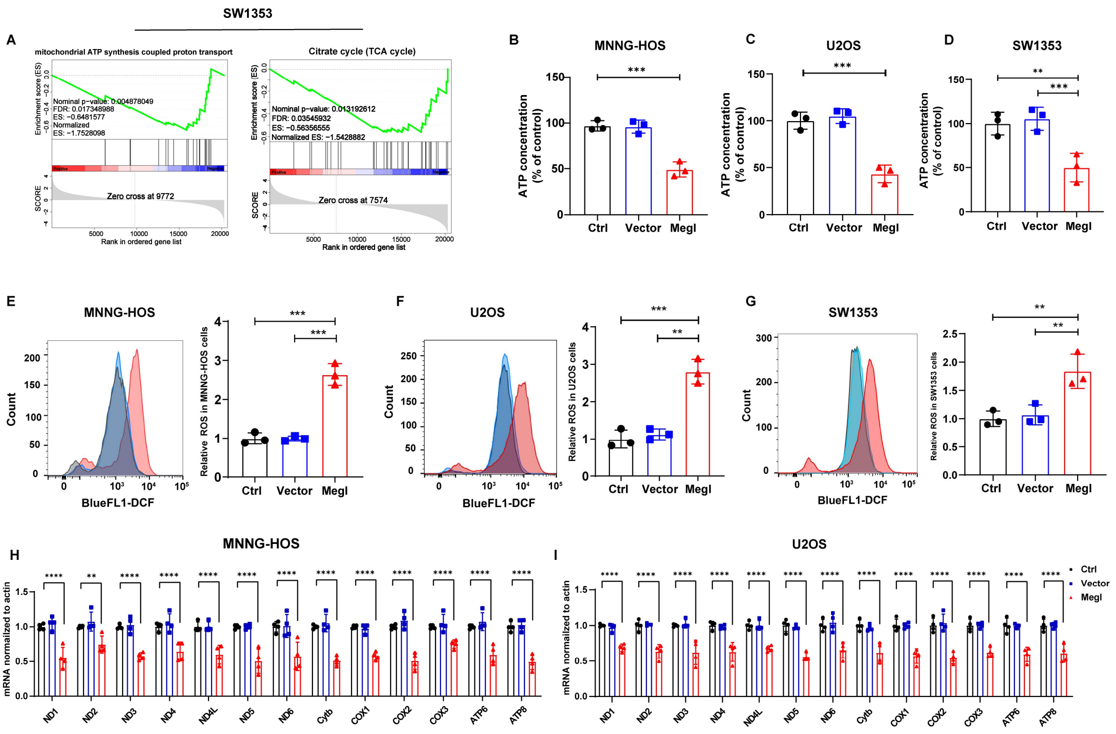

Fig S4. Methionine deprivation induces mitochondrial dysfunction in chondrosarcoma cells. **A.** GSEA analysis of mitochondrial ATP synthesis coupled proton transport pathways and tricarboxylic acid cycle (SW1353) following *L-methioninase* (Megl) overexpression. **B-D** ATP level was determined in the MNNG-HOS, U2OS, SW1353 cells following *L-methioninase* overexpression (n = 3). **E-G.** Left panel: flow cytometry analysis of DCFH-DA staining of MNNG-HOS, U2OS, SW1353 cells treated with *L-methioninase* (Megl) expression; right panel: quantification of relative ROS fluorescence intensity (n = 3). **H-J.** Quantitative PCR assay of expression of mitochondrial genes in MNNG-HOS and U2OS cells following *L-methioninase* (Megl) overexpression (n = 4). Data shown as mean ± SD. In D-F, the p values are derived from one-way ANOVA analysis followed by Tukey's multiple comparison test. In B-G, the p values are derived from one-way ANOVA analysis followed by Tukey's multiple comparison test. The symbols *, **, ***, ****indicate $p < 0.05$, 0.01, 0.001, and 0.0001, respectively.

Fig S5

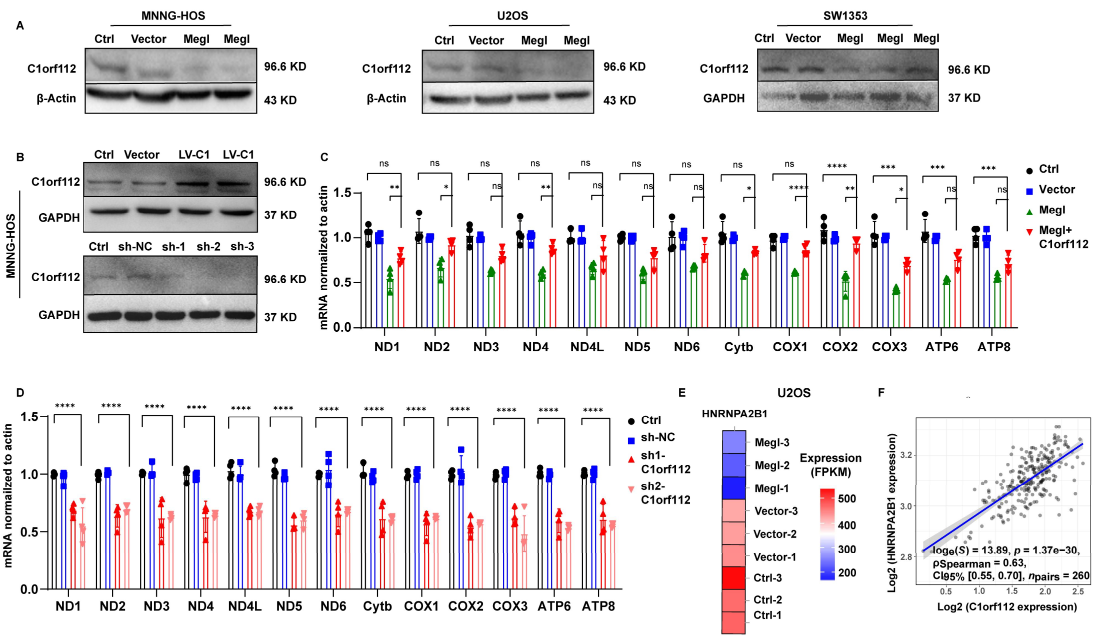

Fig S5. The expression level of C1ORF112 to osteosarcoma cell mitochondrial function

A. Western Blot analysis of the protein expression levels of C1orf112 expression in overexpressing *L-methioninase* MNNG-HOS U2OS, SW1353 cells. **B.** The overexpression and knockdown efficiency of C1ORF112 in the MNNG-HOS cell lines were verified by Western Blot analysis (n=3). Data shown as mean ± SD. **C.** Quantitative PCR of mitochondrial genes after co-overexpression of *L-methioninase* (Megl) and C1ORF112 in MNNG-HOS cells (n=4). **D.** Quantitative PCR of mitochondrial genes in MNNG-HOS cells with C1ORF112 knockout (n=4). **E.** The heatmap of HNRNPA2B1 expression in the osteosarcoma U2OS cells overexpressing the *L-methioninase* (Megl). **H.** HNRNPA2B1 is positively correlated with C1orf112 in TCGA sarcoma cohort. Data shown as mean ± SD. In C-D, the p values are derived from two-way ANOVA analysis followed by Tukey's multiple comparison test. The symbols *, **, ***, ****indicate $p < 0.05$, 0.01, 0.001, and 0.0001, respectively.

Supplemental table 1 – Characteristics of GEO datasets.

<table>
<tr><th rowspan="2">Dataset</th><th rowspan="2">Platform</th><th colspan="3">Sample</th><th rowspan="2">Country</th></tr>
<tr><th>Normal</th><th>Primary tumor</th><th>Metastatic tumor</th></tr>
<tr><td>GSE16088</td><td>Affymetrix Human Genome U133A Array</td><td>6 tissue samples</td><td>14 tissue samples</td><td></td><td>USA</td></tr>
<tr><td rowspan="2">GSE33383</td><td rowspan="2">Illumina human-6 v2.0 expression beadchip</td><td>12 mesenchymal stem cells (MSC)</td><td rowspan="2">84 tissue samples</td><td></td><td rowspan="2">Norway</td></tr>
<tr><td>3 osteoblast (220-ob, 240-OB and Kaat-OB)</td><td></td></tr>
</table>

Supplemental table 2 – oligonucleotides sequences:

Name	Forward	Reverse
C1ORF112 qRT-PCR-Human	GAGGACTGTCAAGCCAAAATTT	AGATATGCTGAACACATCCTGT
Megl-qRT-PCR-Human	GGAGTGATGTGCTTGTGGTGGTC	TGAGGTACTTGGTTGCCGAATGC
ND1 qRT-PCR-Human	CATTCCTAATGCTTACCGAACG	GTAGAGGGTGATGGTAGATGTG
ND2 qRT-PCR-Human	GTTCTACCGTACAACCCTAACA	GTGAATTCTTCGATAATGGCCC
ND3 qRT-PCR-Human	ACAACTCAACGGCTACATAGAA	GTGGCAGGTTAGTTGTTTGTAG
ND4 qRT-PCR-Human	TATTTAGCTGTTCCCCAACCTT	CGTGATAGTGGTTCACTGGATA
ND4L qRT-PCR-Human	GTGCCTATTGCCATACTAGTCT	CGTAGTCTAGGCCATATGTGTT
ND5 qRT-PCR-Human	CTTCTAAACGCTAATCCAAGCC	TAGTGGGCTATTTTCTGCTAGG
ND6 qRT-PCR-Human	ATTTAGGGGGAATGATGGTTGT	CAATAGGATCCTCCCGAATCAA
Cytb qRT-PCR-Human	ATTATGGCTGAATCATCCGCTA	CAGAATGATATTTGGCCTCACG
COX1 qRT-PCR-Human	CAAAGTATTTAGCTGACTCGCC	GAAAGATGAATCCTAGGGCTCA
COX2 qRT-PCR-Human	CGCATCCTTTACATAACAGACG	TAGGAGTTGAAGATTAGTCCGC
COX3 qRT-PCR-Human	CCATAACGCTCCTCATACTAGG	GGTATGTGCTTTCTCGTGTTAC
ATP6 qRT-PCR-Human	CACAGTGATTATAGGCTTTCGC	GAGTAGGCTGATGGTTTCGATA
ATP8 qRT-PCR-Human	TAAATACTACCGTATGGCCCAC	CATTTTGGTTCTCAGGGTTTGT
β-actin qRT-PCR-Human	CCTGTACGCCAACACAGTGC	ATACTCCTGCTTGCTGATCC

Full and uncropped Western blots

Fig.S1G

BCL-2 **β-Actin**

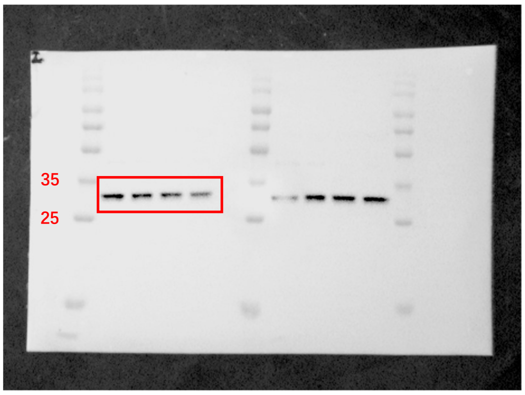

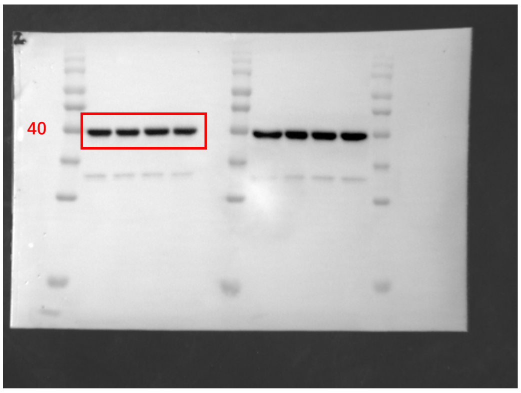

Cleaved-PARP **β-Actin**

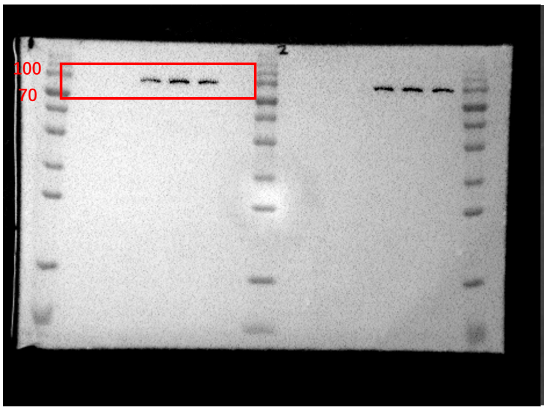

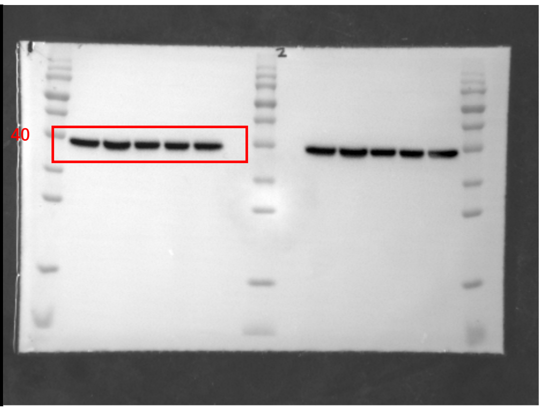

Fig.6A

Met-C1orf112

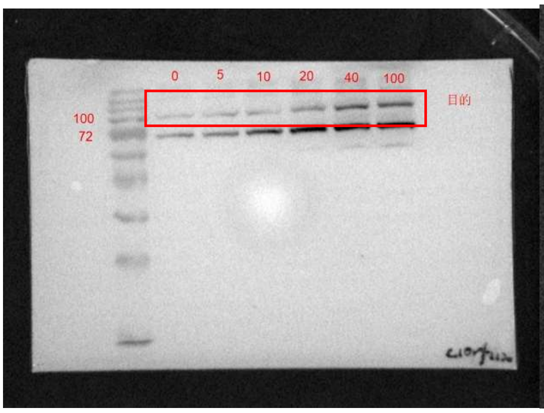

Tubulin

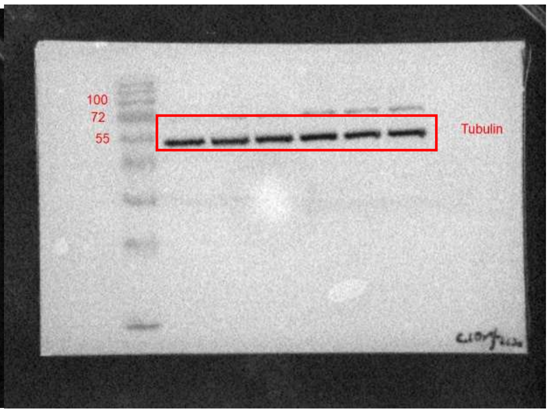

SAM-C1orf112

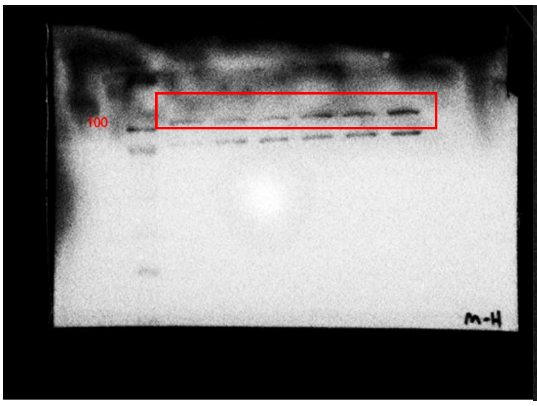

Tubulin

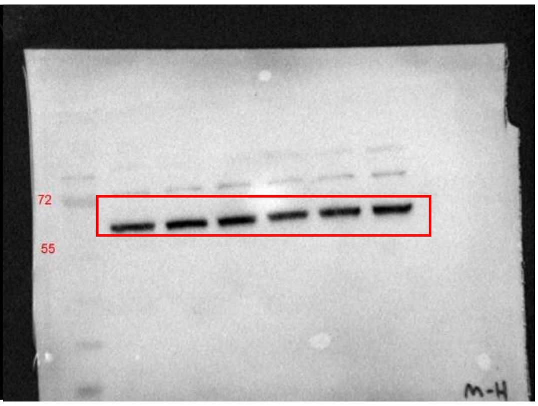

Fig.7F

E-cadherin

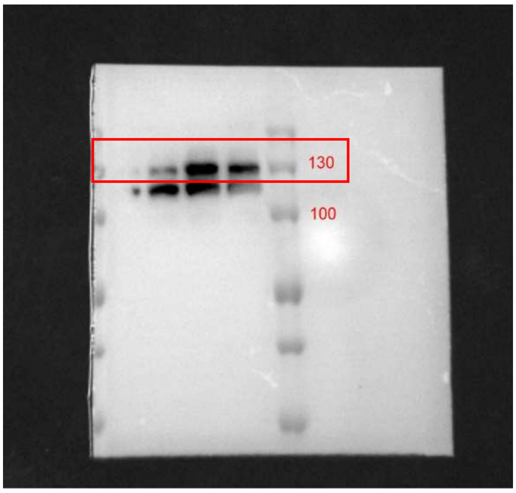

Vimentin

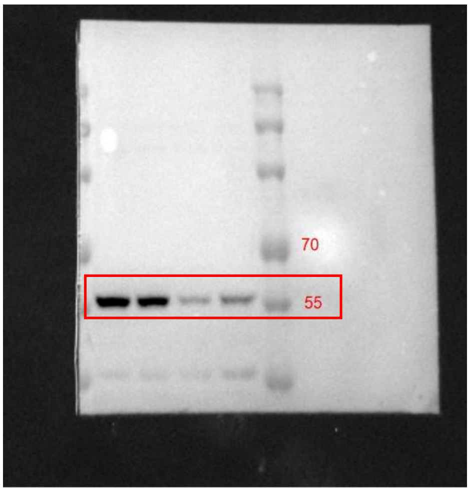

β-Actin

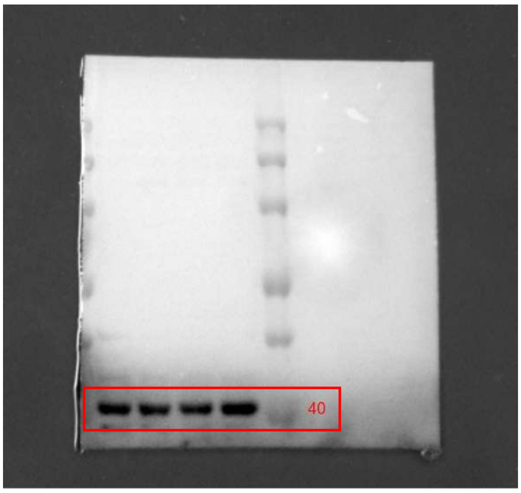

N-cadherin

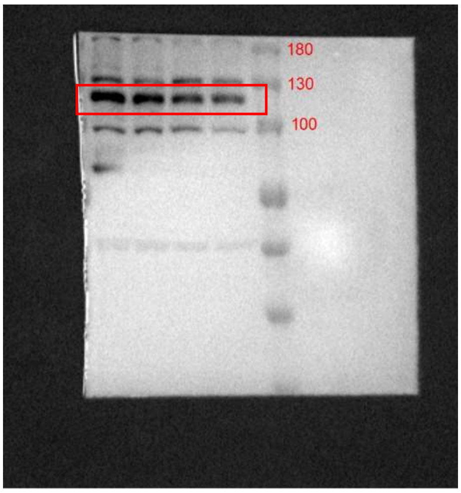

GAPDH

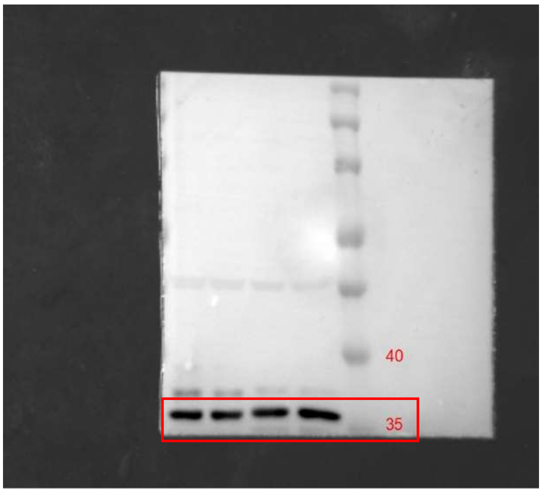

Fig.S5A

MNNG-HOS

C1orf112 β-Actin

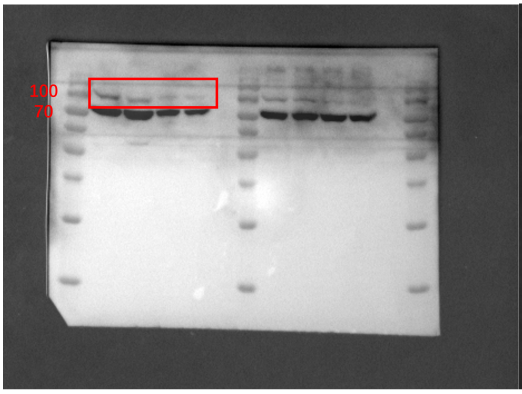

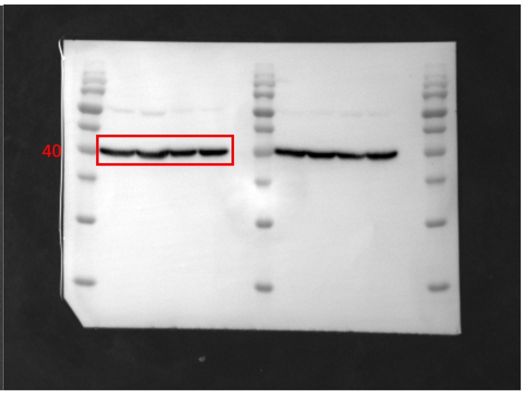

U2OS

C1orf112 β-Actin

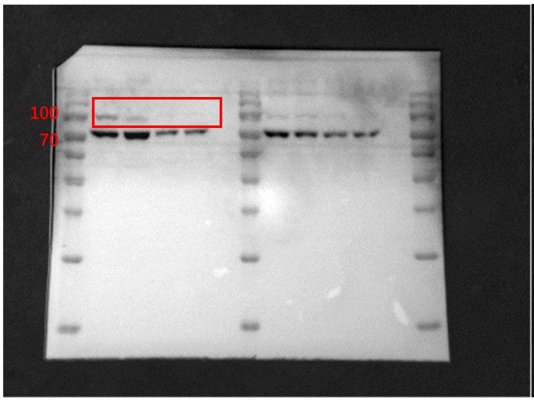

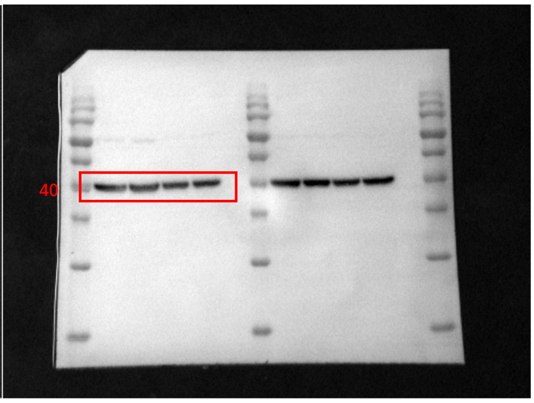

SW1353

C1orf112 GAPDH

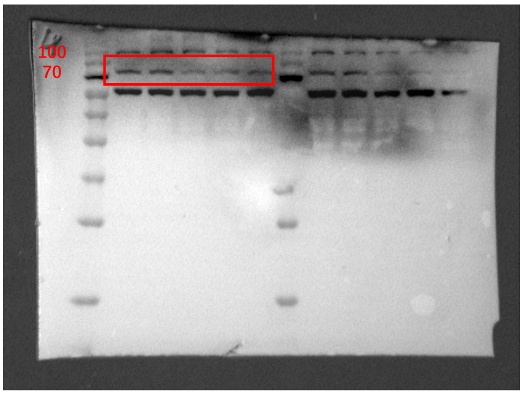

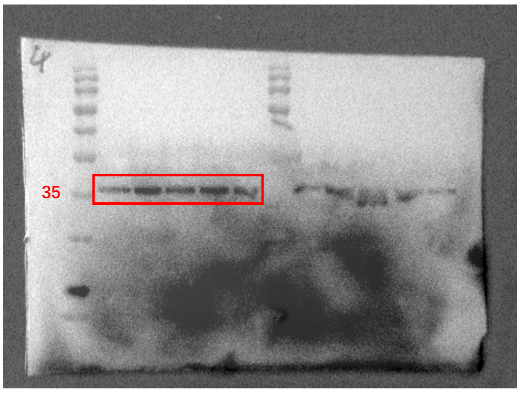

Fig.S5B

lv-C1orf112

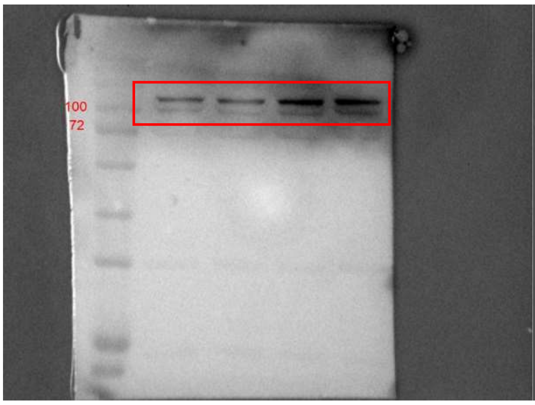

GAPDH

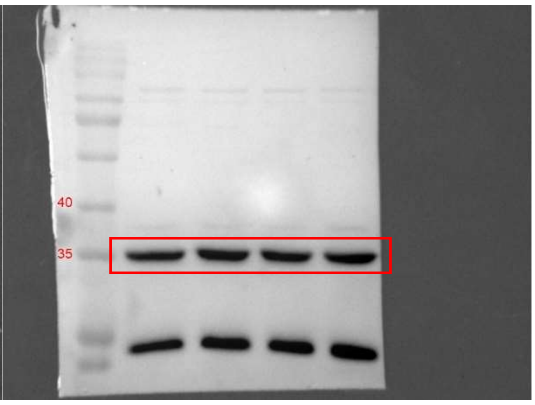

sh-C1orf112

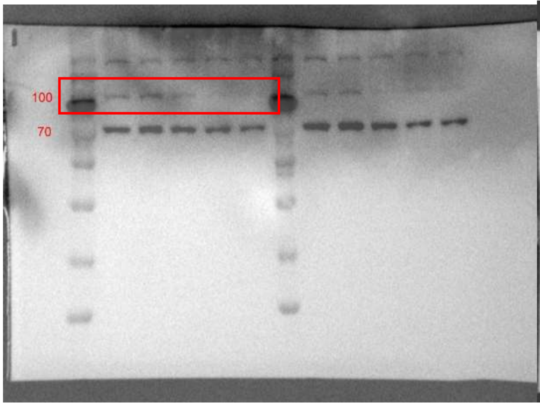

GAPDH

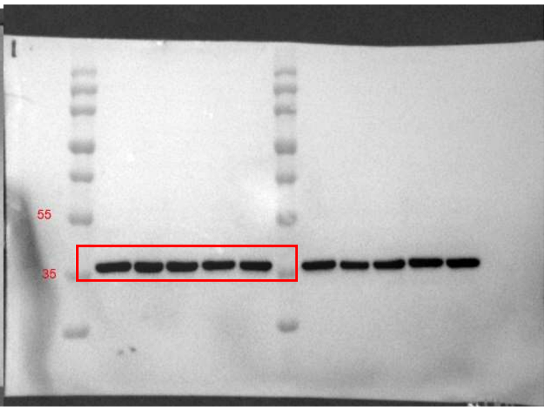

Supplement: Supplementary file 1 — SUPPLEMENTARY MATERIAL [file 41419_2024_6727_MOESM1_ESM.pdf]
